# Mismatch Recognition by *Saccharomyces cerevisiae* Msh2-Msh6: Role of Structure and Dynamics

**DOI:** 10.3390/ijms20174271

**Published:** 2019-08-31

**Authors:** Yan Li, Zane Lombardo, Meera Joshi, Manju M. Hingorani, Ishita Mukerji

**Affiliations:** Molecular Biology and Biochemistry Department, Molecular Biophysics Program, Wesleyan University, Middletown, CT 06459, USA

**Keywords:** Msh2–Msh6, MutSα, DNA repair, fluorescence, Förster resonance energy transfer (FRET), 6-methylisoxanthopterin (6-MI), protein-DNA interactions, induced fit, base dynamics, mismatch repair (MMR)

## Abstract

The mismatch repair (MMR) pathway maintains genome integrity by correcting errors such as mismatched base pairs formed during DNA replication. In MMR, Msh2–Msh6, a heterodimeric protein, targets single base mismatches and small insertion/deletion loops for repair. By incorporating the fluorescent nucleoside base analog 6-methylisoxanthopterin (6-MI) at or adjacent to a mismatch site to probe the structural and dynamic elements of the mismatch, we address how Msh2–Msh6 recognizes these mismatches for repair within the context of matched DNA. Fluorescence quantum yield and rotational correlation time measurements indicate that local base dynamics linearly correlate with *Saccharomyces cerevisiae* Msh2–Msh6 binding affinity where the protein exhibits a higher affinity (*K*_D_ ≤ 25 nM) for mismatches that have a significant amount of dynamic motion. Energy transfer measurements measuring global DNA bending find that mismatches that are both well and poorly recognized by Msh2–Msh6 experience the same amount of protein-induced bending. Finally, base-specific dynamics coupled with protein-induced blue shifts in peak emission strongly support the crystallographic model of directional binding, in which Phe 432 of Msh6 intercalates 3′ of the mismatch. These results imply an important role for local base dynamics in the initial recognition step of MMR.

## 1. Introduction

The mismatch repair (MMR) system ensures the integrity of the genome by scanning, identifying and correcting errors made in DNA when the replication machinery fails. Active MMR reduces the post replicative error frequency by roughly a thousand-fold and prevents recombination between homologous (similar but non-identical) sequences [1,2,3,4]. Many other types of repair processes exist in the cell, such as double strand break repair, non-homologous end joining and base-excision repair; our study is based on the Msh2–Msh6 protein, mainly involved in MMR; thus, we focus on this process. Failure to repair mismatches leads to mutations that persist in the genome. Inactivation of MMR in humans is associated with cancer predisposition, especially Lynch syndrome, and a subset of sporadic tumors in different tissues [5,6,7,8]. MMR, a highly conserved process, has been extensively studied from bacteria to higher order organisms [9,10,11,12,13]. In prokaryotes, MMR is initiated by the MutS homodimer; in eukaryotes, MutS homolog Msh2–Msh6 or MutSα identifies mismatches and small insertion/deletion loops (IDL), while MutSβ or MutS homolog Msh2–Msh3 identifies larger loops. The MutSβ protein is also implicated in the length instability of trinucleotide repeat sequences [14,15]. Upon recognizing a mismatch, MutS changes conformation and recruits MutL in an ATP-dependent manner, and subsequently signals excision of the nascent error-containing strand. Re-synthesis of the excised strand and ligation of the nick completes the repair process.

**Figure 1:**
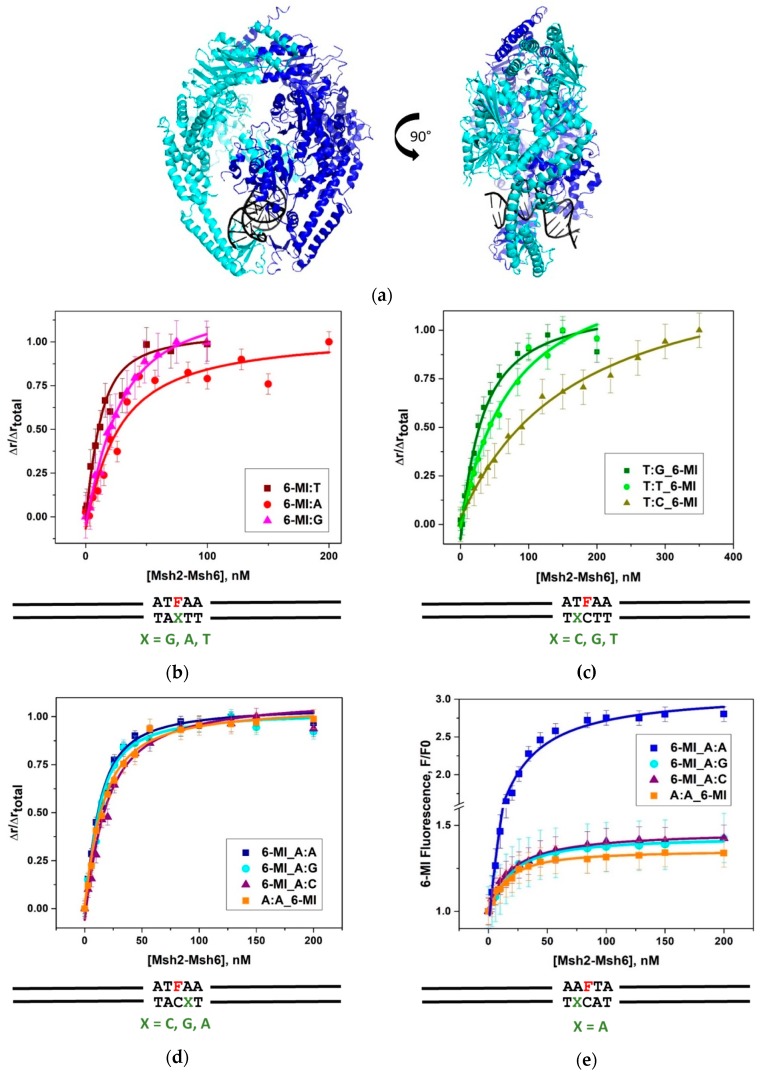
(**a**) Schematic of the Msh2-Msh6 protein in ribbon form showing both front and side views. Msh2 is shown in teal, Msh6 is shown in blue and the DNA is in black. The side view reveals protein-induced bending of the DNA (figure generated from 208B pdb file and Pymol software). Equilibrium fluorescence anisotropy binding curves of Msh2–Msh6 with (**b**) 10 nM 6-MI:X, where X = T, A, G; **(c)** 10 nM T:X_6-MI DNA substrates, where X = C, G, T; (**d**) 10 nM A:X_6-MI DNA where X = C, G, A (DNA sequences in Table S1); (**e**) fluorescence intensity increase of 6-MI_A:X duplexes upon Msh2-Msh6 binding. An intensity increase of approximately 200% is observed when 6-MI is located 5′ to the A:A mismatch. Samples were excited at 340 nm and emission was monitored at 430 nm, as described in the text. Error bars shown are determined from three independent measurements.

The MutS and Msh2–Msh6 protein complexes are θ-shaped dimers that encircle duplex DNA in one of two channels, as observed in the protein-DNA co-crystal structures [16,17,18,19,20]. These crystal structures reveal how the conserved Phe-X-Glu motif from one subunit of MutS or Msh6 of Msh2–Msh6 interacts with the mismatched nucleotide. The Phe residue stacks with the thymine in the G:T mismatch or the unpaired thymine in the +T duplex DNA, while the Glu forms hydrogen bonds with N3 of the thymine, leading to a wider minor groove at the mismatch site (Figure 1a).

A common feature in all of the MutS-mismatched duplex co-crystal structures is the 45–60° protein-induced bend of the duplex DNA at the mismatch site, emphasizing the significance of DNA deformation in mismatch recognition. A series of DNA bending dynamics measurements using atomic force microscopy (AFM) and single-molecule Förster resonance energy transfer (sm-FRET) methods suggest that the kinetics of sampling different bent conformations of MutS-bound mismatched duplex DNA influence mismatch recognition and repair [21,22,23]. The proteins, MutS or Msh2–Msh6, scan the DNA for mismatches or IDLs by sliding along the duplex in a continuous rotational motion during 1D diffusion [24,25,26]. A molecular dynamics (MD) study examined the free-energy profile of DNA bending in the absence of protein and determined a lower free energy cost of ~1.25 kcal/mol to bend duplex DNA containing a mismatch compared with the homoduplex [27]. DNA bending via smooth deformation is observed for homoduplex DNA, whereas for mismatched duplex DNA, bending typically leads to kinking at the mismatch site [21,27].

MutS or Msh2–Msh6 protein complexes must distinguish between multiple types of mismatch or IDL sites from a large excess of canonical base pairs (bp) post replication. These mismatches or IDLs may share some subtle structural and dynamic differences from normal B-form duplex DNA; however, all single base mismatches are not recognized with the same affinity. Several biochemical assays, including electrophoretic mobility shift assays (EMSA) and surface plasmon resonance (SPR) measurements, have been carried out to characterize the binding affinities of *E. coli* MutS, *S. cerevisiae* and human Msh2–Msh6 with various mismatched bps [28,29,30]. These studies suggest that duplex DNA substrates containing G:T and +T are well-recognized by MutS/Msh2–Msh6, while the C:C mismatched bp is weakly recognized. Mismatch repair efficiencies measured in reconstituted *E. coli* methyl-directed MMR systems and cell-free extracts of human neuroblastoma and fibroblast cells correlate with the affinity data and indicate that the G:T mismatch is repaired with the highest efficiency and C:C with the least, highlighting important biological consequences of the differences in mismatch recognition and binding affinity [31,32,33]. To identify the elements that lead to these differences in mismatch recognition and binding affinity, we investigated possible correlations among the following parameters: DNA duplex flexibility in the presence of a mismatch, dynamics of the mismatched bp, Msh2–Msh6 binding affinity and the efficiency of repair.

Fluorescence and nuclear magnetic resonance (NMR) measurements of the local dynamics and flexibility of mismatched bp have pointed to their relative importance in mismatch recognition [34,35]. NMR measurements of the internal dynamics of G:T and G:C bps in duplex DNA revealed a difference in structural dynamics between the mismatched site and the canonical bp [34]. Time-resolved fluorescence spectroscopic measurements of the motional dynamics of the fluorescent analogue 2-aminopurine (2-AP) incorporated next to a G:T, +T or C:C site in duplex DNA revealed enhanced dynamics of 2-AP immediately neighboring the mismatch or IDL site, which dissipated with increasing distance from the site [35]. These fluorescence measurements along with the NMR and MD results suggest that the region near the mismatch or IDL site experiences greater dynamic motion [27,30,35,36,37], which may be an important feature in recognition [38].

To examine the base-specific dynamics of a broad set of mismatched bps, we have incorporated a fluorescent guanosine analogue 6-methylisoxanthopterin (6-MI) at or next to seven different mismatch sites in this study (Table S1). Since the hydrogen bonds between 6-MI and cytosine mimic those of a canonical G:C bp, 6-MI incorporation minimally disrupts the conformation of duplex DNA [39]. Furthermore, 6-MI emits duplex-enhanced fluorescence when incorporated into the ATFAA pentamer sequence (F = 6-MI), and the fluorescence is sensitive to local perturbations in DNA structure at a single base level [40]. The binding affinities of *S. cerevisiae* Msh2–Msh6 with 6-MI-containing mismatched duplex DNA substrates were measured and found to linearly correlate with increased dynamics as measured by the decay lifetimes, quantum yields and the local rotational times. From these results, we infer that the Msh2–Msh6 heterodimer binds mismatched substrates with higher affinity if the mismatch site exhibits significant dynamic motion as measured by 6-MI. These binding results affirm the importance of local flexibility that may be exploited by MutS proteins in mismatch recognition. We further investigated if binding affinity correlates with induced bending, as measured by steady-state and time-resolved FRET experiments, and determined that the degree of bending is similar for all mismatched substrates. Lastly, Msh2–Msh6 binding leads to decreased dynamics and spectral blue shifts of 6-MI, which indicate a stabilization effect at the mismatch site, consistent with co-crystal structures where the Phe-X-Glu motif of Msh6 specifically interacts with the mismatched base or IDL site [19]. Significantly, this comprehensive investigation elucidates the factors governing initial mismatch recognition by *S. cerevisiae* Msh2–Msh6, provides solution validation of structural features from X-ray crystallography and reports the novel finding that binding affinity linearly correlates with local dynamics of the mismatch and not bending propensity.

## 2. Results

### 2.1. Msh2-Msh6 Recognizes Mismatched DNA Base Pairs with Different Affinities

This study investigates specific properties of mismatched DNA substrates to determine those elements that influence recognition and repair through examination of *S. cerevisiae* Msh2–Msh6 binding affinity, global DNA bending and local DNA base dynamics of mismatched duplexes with and without protein bound. The DNA substrates are a series of 34-bp duplexes each containing one of seven different single base mismatches (all except C:C); to reduce variability due to sequence context, we inserted the mismatches into the same or similar pentamer sequence (Table S1).

We initially measured Msh2–Msh6 binding to a subset of the mismatched substrates (G:T, G:A and +T) using electrophoretic mobility shift assays (EMSA) (Figure S1). The mismatched substrates exhibited two sets of bands, where the faster migrating band was attributed to the specific Msh2-Msh6-mismatched DNA complex while the slower one was attributed to non-specific binding [28]. As expected we observe that Msh2–Msh6 has the same affinity for the G:T and +T substrates within error, but has two-fold weaker affinity for the G:C substrate (Table S2). Binding to the G:A duplex is weaker than G:T but stronger than G:C as observed previously by EMSA [29].

We further investigated Msh2–Msh6 binding using fluorescence spectroscopy and 6-MI incorporated DNA duplexes, which allowed us to monitor both binding affinity and local base-specific dynamics. To determine whether Msh2–Msh6 recognizes a 6-MI-containing base pair as a mismatch, we compared Msh2–Msh6 binding affinity for the same duplexes containing either 6-MI or guanine (the latter 5′-end-labeled with Alexa Fluor 647). These fluorescence anisotropy binding assays yielded virtually identical affinities, indicating the 6-MI probe is not recognized as a mismatch by Msh2–Msh6 (Figure S2a). Msh2–Msh6 binds duplexes with 6-MI:C or G:C with the same five- to eight-fold weaker affinity relative to duplexes containing an unpaired thymine (+T) with an adjacent 6-MI:C or G:C (Figure S2b).

Using fluorescence anisotropy we measured Msh2–Msh6 binding to the full set of seven mismatched duplexes containing 6-MI paired with either thymine, adenine or guanine (6-MI:T, 6-MI:A or 6-MI:G) (Figure 1b,e and Table 1). The high binding affinity of Msh2–Msh6 observed for the 6-MI:T containing duplex (13.7 ± 3.4 nM) is consistent with previously reported *K*_D_ values of approximately 4 nM or less [30]. Msh2–Msh6 also displays high binding affinity for the 6-MI:A and 6-MI:G mismatched duplexes with *K*_D_ values of 25.8 ± 6.9 nM, and 10.4 ± 5.8 nM, respectively (Table 1 and Figure 1b). As expected, binding to matched 6-MI:C duplex DNA is significantly weaker, but still measurable by fluorescence anisotropy with a *K*_D_ value of 121.5 ± 12.1 nM (Figure S2a). Importantly, for the G:T, +T and G:A duplexes, the EMSA and fluorescence binding data exhibit the same general trends, where Msh2–Msh6 binds more tightly to the 6-MI:T and +T_6-MI duplexes relative to the 6-MI:A duplex and weakest to 6-MI:C (Figure S1b and Figure 1b–e).

The solution measurements give approximately two- to three-fold lower *K*_D_ values relative to the EMSA measurements and show a bigger difference in affinity between mismatched and homoduplex DNA [28]. Given that samples in the gel can be subject to dilution and caging effects, it is not surprising that the two methods yield slightly different *K*_D_ values [41,42].

The relative affinities of Msh2-Msh6 for T:X containing mismatched DNA duplexes were measured with 6-MI located 3′ to the mismatched thymine (Figure 1c and Table 1). These duplexes are hereafter referred to as T:X_6-MI to indicate both the type of mismatch and the 5′ location of the mismatch relative to the 6-MI probe. As expected, Msh2–Msh6 binds the T:G_6-MI mismatched duplex DNA with high affinity giving a Κ_D_ of 25.9 ± 1.2 nM (Table 1 and Figure 1b). This *Κ*_D_ value is approximately two-fold higher than our measured values for 6-MI:T and the end-labeled G:T duplex DNA. This difference might be due to a sequence context effect, where up to two- to three-fold difference in binding affinity has been observed for the same mismatch with different nearest neighbor nucleotides [28,30]. We note that in going from 6-MI:T to T:G_6-MI the 3′ bases change from two purines to purine and pyrimidine, which could be a source of the difference in binding affinity [30]. Additionally, changing the orientation of the mismatch can also affect binding affinity, as has been reported previously [43]. In contrast, Msh2-Msh6 binds weakly to two mismatched duplexes, T:T_6-MI and T:C_6-MI, with Κ_D_ values of 77.5 ± 20.0 and 112.6 ± 18.7 nM (Table 1 and Figure 1c).

Within the ATFAA sequence used in this study, Msh2-Msh6 binds with high affinity to A:X type mismatches as determined by fluorescence anisotropy. Duplexes containing A:X mismatches are referred to as 6-MI_A:X indicating the type of mismatch and the location of the mismatched bp 3′ to the 6-MI probe. Upon Msh2-Msh6 binding *K*_D_ values of 7.9 ± 2.0, 9.0 ± 2.8 and 13.3 ± 3.6 nM were obtained for the 6-MI_A:A, 6-MI_A:G and 6MI_A:C DNA duplexes, respectively (Figure 1d and Table 1). The *K*_D_ values determined from the 6-MI fluorescence intensity increase observed upon protein binding are in good agreement with those measured by fluorescence anisotropy, indicating that a change in probe intensity, typically associated with DNA bending or deformation, accompanies protein binding (Figure 1e and Table 1). To assess whether probe location influences binding affinity, we generated a duplex DNA construct A:A_6-MI, where the A:A mismatch is incorporated 5′ to 6-MI:C in an AAFTA pentamer sequence. We measured *K*_D_ values of 11.3 ± 3.1 and 10.4 ± 2.4 nM for this construct by fluorescence anisotropy and fluorescence intensity, respectively. These values are comparable to the values of 7.9 ± 2.0 and 19.1 ± 3.5 nM, respectively, obtained for the 6-MI_A:A duplex indicating Msh2–Msh6 binds the A:A mismatch with high affinity, independent of 6-MI probe location (Figure 1d,e).

### 2.2. 6-MI Fluorescence Reveals Significant Variations among Mismatched Base Pairs

**Figure 2:**
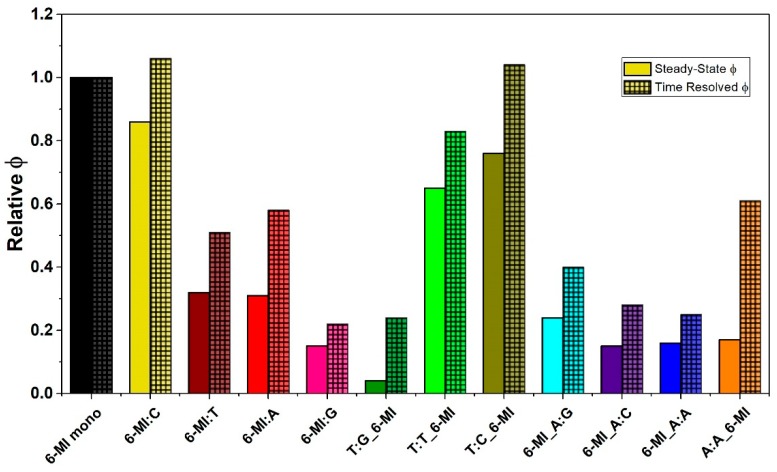
Steady-state and time-resolved 6-MI quantum yields of the mismatched DNA duplexes. The steady-state (ϕ_relSS_) and time-resolved quantum yields (ϕ_relTR_) are reported relative to 6-MI monomer quantum yield, which is normalized to 1. Values are given in Table S3. Steady-state quantum yields are shown in solid bars and time-resolved quantum yields are shown in hatched bars. T:T_6-MI, T:C_6-MI and 6-MI:C exhibit the highest quantum yields signifying a lack of dynamic quenching.

We examined the steady-state quantum yield (ϕ_relSS_) of 6-MI in the ATFAA pentamer sequence with a mismatched bp introduced either at the 6-MI site or next to it. All steady-state quantum yields are reported relative to 6-MI monomer (ϕ = 0.70) [44,45]. For all duplex substrates examined, 6-MI:C exhibits the highest ϕ_relSS_ of 0.86, indicating the least amount of fluorescence quenching occurs when 6-MI is paired with cytosine [40] (Figure 2 and Table S3). The other 6-MI:X (X = T, A, G) mismatched duplexes showed a significant reduction in steady-state quantum yield (0.15–0.32), which suggests an increased amount of quenching of the 6-MI excited state in these duplexes (Figure 2). In the ATFAA sequence context, increased quenching is consistent with greater mobility of the probe [46].

Similarly, the steady-state quantum yield of 6-MI is relatively low when it is adjacent to different mismatches in either the 3′ or 5′ position. When 6-MI:C is located 3′ to a T:G mismatch, a ϕ_relSS_ value as low as 0.04 is observed, suggesting that this location with respect to the T:G mismatch leads to a high degree of quenching of 6-MI (Figure 2). The A:A_6-MI duplex also experiences quenching and exhibits a relatively low ϕ_relSS_ There are two outliers from this trend, the T:T_6-MI and T:C_6-MI duplexes exhibit a moderate ϕ_relSS_ of 0.65 and 0.76, respectively. These moderate quantum yields are indicative of a reduced amount of collisional quenching of the excited state of 6-MI, consistent with the 6-MI being in a relatively rigid duplex context [46]. Thus, these results suggest that the local environment and mobility of 6-MI adjacent to T:T and T:C bps differs from when it is adjacent to a T:G mismatch.

All three duplex DNA molecules with 6-MI:C located 5′ to the A:X mismatch exhibited relatively low quantum yields, with ϕ_relSS_ values of 0.16, 0.24 and 0.15 respectively for 6-MI_A:A, 6-MI_A:G and 6-MI_A:C. As discussed above, these relatively low quantum yields indicate increased quenching of the 6-MI probe, consistent with increased flexibility of the probe and the local environment similar to what we observed for the A:A_6-MI and T:G_6-MI duplexes.

The time-resolved quantum yield (ϕ_relTR_) values determined from time-resolved fluorescence decay measurements exhibit a similar trend as the steady-state quantum yield (ϕ_relSS_) values (Figure 2 and Table S3). Specifically, the T:T _6-MI and T:C_6-MI DNA duplexes exhibit moderately high ϕ_relTR_ values of 0.83 and 1.04, respectively, which are significantly higher than the other mismatched DNA substrates. These relatively high ϕ_relTR_ values are comparable to that obtained for the 6MI:C duplex, which suggests that the local environment of 6-MI:C in the T:C or T:T mismatched duplex is similar to that in a matched homoduplex (Table S3). The other mismatched duplexes all exhibit ϕ_relTR_ values ≤0.6, consistent with a significant amount of dynamic quenching relative to the matched DNA. The ϕ_relTR_ values for all the mismatch-containing substrates are higher than the corresponding steady-state quantum yields, indicating both static and dynamic quenching of the 6-MI probe is occurring [47].

#### Local Structure and Dynamics of Mismatched Base Pairs

**Figure 3:**
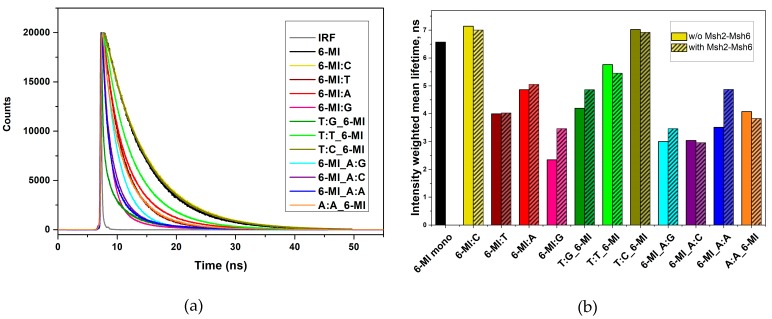
Base-specific investigation of mismatched base pairs using 6-MI fluorescence excited-state decay curves and intensity-weighted mean lifetimes (**a**) The instrument response function is shown in gray, and monomeric 6-MI fluorescence decay (black) is shown as a reference. The 6MI:C (yellow) and T:C_6-MI (dark yellow) duplex DNA substrates exhibit fluorescence decays similar to 6-MI monomer, indicative of significantly reduced quenching, which is further confirmed by the absence of a short-lived component (Table S4). All curves were obtained with excitation at 375 nm and emission at 460 nm. (**b**) Intensity-weighted mean lifetimes were derived from the time-resolved fluorescence decays shown in panel a. Shorter lifetimes are observed for the Msh2-Msh6 high-affinity substrates; which exhibit the same or longer mean lifetimes upon protein binding. 6-MI monomer is shown as a reference (black).

We examined the excited-state lifetimes of the 6-MI probe in different mismatch contexts (Table S1) to further investigate how mismatch incorporation alters the local DNA structure (Figure 3). The fluorescence decay of the 6-MI monomer is well-described by a single lifetime of 6.57 ns, as reported previously [40,48,49]. When 6-MI is incorporated into the ATFAA sequence and paired with dC in a homoduplex, two lifetimes are observed: a mid-range (3.47 ns) one and a longer one (7.33 ns) similar to that of 6-MI monomer (Table S4). Incorporation of 6-MI within or next to a mismatch site in the ATFAA sequence alters the decay kinetics and adds a short lifetime τ_1_ (0.30–1.38 ns), leading to three distinct lifetimes. The shortest lifetime τ_1_ potentially reflects a quenched population. The subpopulation associated with the longest lifetime τ_3_ is thought to arise from a constrained conformation of the probe in this sequence context. The subpopulation associated with the intermediate lifetime τ_2_ (2.09–4.04 ns) is attributed to an intermediate conformation between the two states (Table S4) [46].

We compared the relative dynamics of the different mismatches through the mean lifetime of the 6-MI probe when directly incorporated within a mismatch (6-MI:X, where X = T, A, G) (Figure 3 and Table S4). The mean lifetime of 6-MI incorporated at a mismatch (2.34–4.86 ns) is significantly shorter than that of 6-MI monomer (6.57 ns) or of a 6-MI:C bp in homoduplex DNA (7.14 ns). The shorter mean lifetimes observed for the 6-MI mismatched bps result from higher subpopulations of the shortest lifetime component and is attributed to stronger collisional quenching at the mismatch site, most likely caused by increased interactions with adjacent bases. The fluorescence decay of the T:G_6-MI mismatched duplex also exhibited three excited-state lifetimes similar to duplexes with 6-MI located at the mismatch site (Table S4). Interestingly, 6-MI_A:X (X = G, C, A) duplexes exhibit larger subpopulations of the shortest and the intermediate lifetimes, and a smaller fractional subpopulation of the longest lifetime (α_3_ = 0.04–0.08), resulting in a relatively short mean lifetime (3.00–3.51 ns) compared to the other mismatch-containing duplexes (Figure 3 and Table S4). These results suggest that a large amount of quenching of 6-MI fluorescence occurs in this mismatch context. On the other hand, the A:A_6-MI duplex with 6-MI incorporated 3′ of the A:A mismatch in the AAFTA sequence (Table S1) exhibits a slightly longer mean lifetime (4.07 ns vs. 3.51 ns for 6-MI_A:A), demonstrating a subtle difference in 6-MI quenching dynamics depending on probe location relative to the A:A mismatch site.

In contrast, the T:C_6-MI mismatched duplex exhibits fractional subpopulation distributions and lifetimes that are similar to the 6-MI:C homoduplex DNA (Figure 3 and Table S4). This result is consistent with our finding that the T:C_6-MI duplex has relatively high steady-state and time-resolved quantum yields that are comparable to the 6-MI:C duplex, and collectively these results suggest that 6-MI does not experience significant dynamic quenching when adjacent to a T:C mismatch. Similarly, the fluorescence decay of the T:T_6-MI duplex yields two fractional subpopulations associated with the intermediate lifetime (α_2_ = 0.34) and the longest lifetime (α_3_ = 0.66). 

The disappearance of the subpopulation with the shortest lifetime suggests a reduction in dynamic quenching of 6-MI when adjacent to the T:T mismatch. Previously, we determined that 6-MI exhibited duplex-enhanced fluorescence in the ATFAA sequence context as duplex formation significantly reduces the amount of dynamic quenching by structurally constraining the probe. Introduction of a mismatch relaxes the structural constraint and increases dynamic quenching [40,46]. For the T:T and T:C mismatched duplexes the observation of excited state dynamics comparable to homoduplex DNA implies that T:T and T:C do not relax the structural constraint of the duplex in the same manner as the other mismatched base pairs (Figure 3).

**Figure 4:**
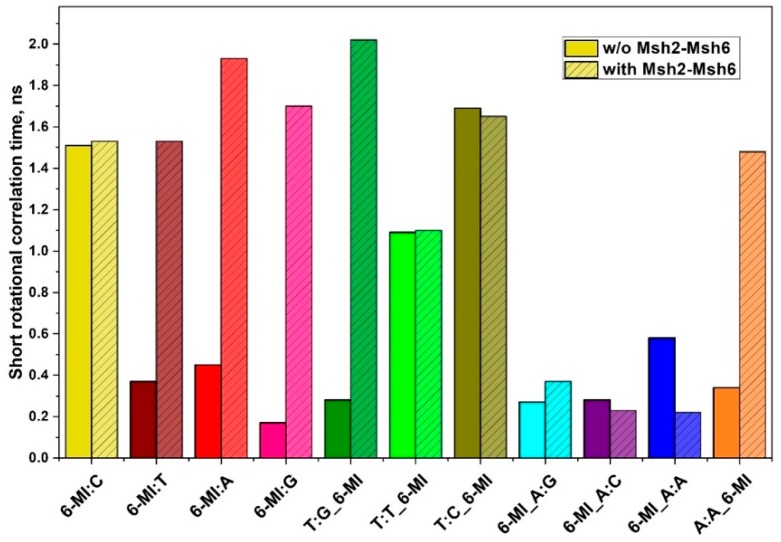
Time-resolved anisotropy decays of 6-MI-containing duplexes with and without Msh2–Msh6. A faster rotational correlation time corresponding to local motion (*θ*_L_) is observed in the Msh2–Msh6 high affinity substrates without protein (solid bars). Upon Msh2–Msh6 binding, an increase in *θ*_L_ for 6-MI at a mismatched bp or in the T:G_6-MI and A:A_6-MI substrates is attributed to local stabilization of the probe. No changes upon protein binding are observed in the 6-MI:C, T:T_6-MI and T:C_6-MI substrates. Data were collected and analyzed as described in the text.

To monitor the mobility of the 6-MI probe within the different mismatched substrates directly we performed time-resolved fluorescence anisotropy measurements in the absence and presence of Msh2–Msh6 (Figure 4 and Table S5). Similar to previous studies using either 6-MI or 2-AP fluorescent probes, the anisotropy decays are well described by a two-component exponential decay model consisting of a relatively short (1–5 ns) and a long rotational correlation time (15–25 ns) [35,40]. The shorter rotational correlation time *θ*_L_ represents the local motion of the 6-MI probe, whereas the longer rotational correlation time *θ*_R_ is associated with the global tumbling motion of the duplex DNA.

In the case of the 6-MI:C containing homoduplex the local rotational correlation time *θ*_L_ of the 6-MI probe is 1.51 ns (Table S5 and Figure 4), consistent with previously reported values for this substrate [40]. This relatively long *θ*_L_ is indicative of reduced rotational mobility of the 6-MI presumably due to H-bonding with cytosine and base stacking interactions. In distinct contrast the three mismatched duplexes (6-MI:X, X = T, A, G) have much shorter *θ*_L_ values (0.37, 0.45, and 0.17 ns respectively), which is comparable to the rotational correlation time observed for 6-MI incorporated into a single stranded ATFAA sequence (0.32 ns) [40]. The relatively fast correlation times observed (<0.5 ns) indicate the 6-MI probe experiences a considerable degree of dynamic motion in the mismatched duplexes similar to that of ssDNA.

Even when located adjacent to a mismatch, the 6-MI probe reports on the dynamic motion of the mismatch, indicating 6-MI sensitivity extends to the local area of the mismatch. Thus, when 6-MI is located 3′ to the T:G mismatch (T:G_6-MI), a fast rotational correlation time *θ*_L_ of 0.28 ns is measured, indicative of a large amount of motional dynamics of 6-MI (Table S5 and Figure 4), similar to that observed in the 6-MI:X mismatch duplexes. The T:T_6-MI duplex DNA exhibits a rotational correlation time *θ*_L_ of 1.09 ns and similarly, a significantly longer *θ*_L_ of 1.69 ns is measured for the T:C_6-MI duplex. These longer values are similar to that observed for the 6MI:C duplex DNA, suggesting that 6-MI is in a constrained geometry in these two substrates, just as in the homoduplex. In contrast, enhanced dynamics are observed when the 6-MI probe is located 5′ to all three A:X (X = G, C, A) mismatch sites, with *θ*_L_ values in the range of 0.27–0.58 ns. Significantly, the relative mobility of the 6-MI probe does not depend on its location relative to the mismatch. This is shown by a comparison of the A:A_6-MI and 6-MI_A:A duplexes, where 6-MI is located either 3′ or 5′ of the A:A mismatch and the *θ*_L_ values are approximately the same, 0.34 and 0.58 ns respectively (Figure 4 and Table S5).

### 2.3. Msh2-Msh6 Binding Affects Mismatched Base Pair Dynamics

To determine the effects of Msh2–Msh6 binding on the mismatched bp dynamics, we measured the 6-MI fluorescence intensity decays of all the DNA substrates in the presence of protein (Figure 3). Protein binding led to both subtle increases and decreases in 6-MI mean lifetime with no clear trends. For some of the mismatched duplexes (6-MI:A, 6-MI:G, T:G_6-MI, 6-MI_A:G and 6-MI_A:A) we observed a shift or redistribution of subpopulations to longer-lived components, leading to an increase in mean lifetime upon binding (Figure 3 and Table S4). Interestingly, the T:T_6-MI, T:C_6-MI and 6-MI:C duplexes responded similarly to Msh2-Msh6 binding and exhibited a slight *decrease* in mean lifetime (Δτ_m_ = 0.1–0.3 ns). Generally, these changes were smaller than those observed for the other mismatched duplexes (Table S4).

To further probe the effect of protein binding on DNA dynamics and conformation, we determined the local (*θ*_L_) and global (*θ*_R_) rotational correlation times using 6-MI anisotropy. The protein-induced changes were larger and exhibited clear trends with respect to mismatch type. For all of the duplexes examined, a significant increase in the global rotational time, *θ*_R_, was observed upon protein binding (Table S5). This *θ*_R_ increase is attributed to an increase in the size, mass and hydrodynamic volume as a consequence of protein-DNA complex formation, confirming the presence of the complex.

For all the 6-MI:X mismatched duplexes examined, the local motion component *θ*_L_ increased several fold (1.53–1.93 ns) upon Msh2-Msh6 binding, indicating that protein binding stabilizes the 6-MI probe in the mismatched bp. A similar effect is observed for the T:G_6-MI duplex substrate, where protein binding leads to an almost 10-fold increase in *θ*_L_, from 0.28 to 2.02 ns. Interestingly, the dynamics of 6-MI in the T:T_6-MI and T:C_6-MI duplexes remain relatively constant upon protein binding and in fact, decrease slightly, consistent with the slight reduction in mean lifetime. This behavior is similar to that observed for the homoduplex and very different from the behavior of the other mismatched duplexes (Figure 4). For all three duplexes (6-MI:C, T:T_6-MI and T:C_6-MI), the probe is already structurally constrained as shown by the relatively long *θ*_L_ (>1 ns) obtained in the absence of protein. From these data we infer that protein binding releases some of the structural constraints leading to slightly shorter lifetimes and rotational correlation times.

In the case of the 6-MI_A:X mismatched duplexes, protein binding does not lead to a longer *θ*_L_, possibly because of the placement of the probe 5′ to the mismatch, the effects of protein binding are not detected. To assess whether the location of the probe influences the degree of stabilization observed, we compared *θ*_L_ of the 6-MI_A:A and A:A_6-MI mismatched duplexes and found that for A:A_6-MI, with the probe on the 3′ side of the mismatch, protein binding leads to a roughly five-fold increase in *θ*_L_. Importantly, this difference in behavior is only observed upon protein binding as the anisotropy decays of A:A_6-MI and 6-MI_A:A yield similar *θ*_L_ values without protein, suggesting that in DNA alone the 6-MI probe is relatively mobile in both locations.

### 2.4. Msh2-Msh6 Preferred Binding Orientation and Phe Intercalation Reported by 6-MI Fluorescence

**Figure 5:**
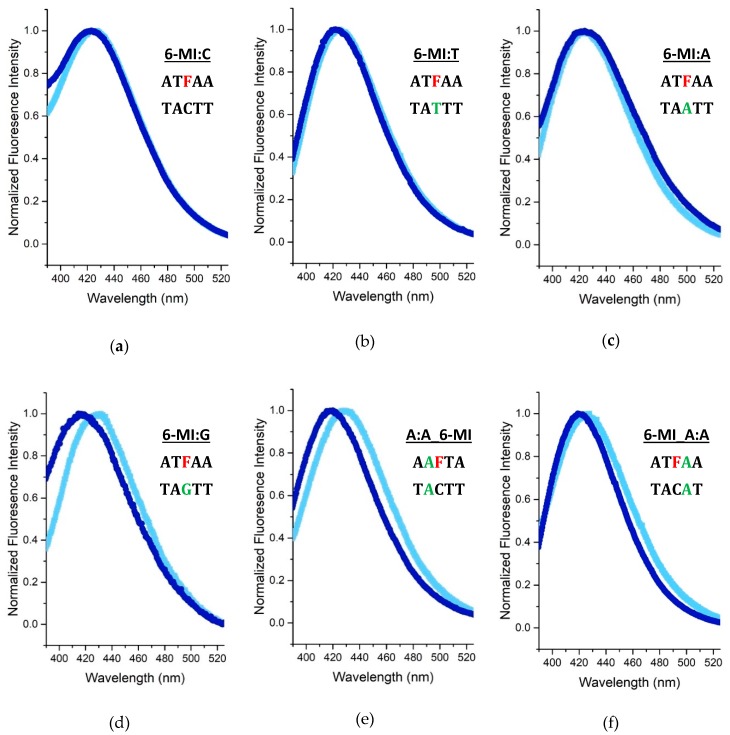
6-MI Spectral Blue Shifts Report on Msh2-Msh6 Preferred Binding Orientation and Phe Intercalation. The 6-MI emission spectra of mismatched DNA duplexes with (dark blue) and without (light blue) Msh2-Msh6 protein. Protein binding induces a shift to shorter wavelengths for some substrates. (**a**–**c**) The 6-MI:C, T, and A substrates show no shift in emission. (**d**,**e**) The 6-MI:G and A:A_6-MI substrates show a significant blue shift in emission after addition of Msh2-Msh6 (**f**) the 6-MI_A:A duplex exhibits a –5 nm shift in peak emission. All spectra were obtained with 20 nM DNA; for spectra with Msh2–Msh6, sufficient protein was added to ensure >90% protein-bound DNA.

The 6-MI emission spectra of the mismatch-containing duplexes report on the local environment of the probe, providing some insight into the nature of the protein interaction. We find that the 6-MI:T, 6-MI:A and 6-MI:C duplexes do not exhibit any changes in spectral emission upon protein binding. Conversely, we find that the 6-MI:G, A:A_6-MI and 6-MI_A:A substrates all exhibit a shift in peak emission to shorter wavelengths upon protein binding, with the largest shifts observed for 6-MI:G and A:A_6-MI (Figure 5). We attribute any blue shifts in the 6-MI emission spectra to an increase in hydrophobicity of the local environment, possibly reflecting stacking interactions with the conserved Phe 432 residue of the Phe-X-Glu motif that contacts the mismatch. As expected, no shift is observed for the 6-MI:C homoduplex DNA as there is no mismatch site and no prolonged interaction with the protein (Figure 5a). In the case of the 6-MI:T and 6-MI:A DNA duplexes (Figure 5b,c), we speculate that the Phe preferentially stacks with the thymine or adenine base in these asymmetric mismatches as reported previously from solution studies [43] and observed in Msh2–Msh6 co-crystal structures with G:T or C:A mismatches (Figure 1) [18]. The relatively large 10 nm spectral blue shift observed with the 6-MI:G duplex is consistent with an increase in hydrophobicity in the local environment potentially from a stacking interaction between Phe 432 and the 6-MI probe (Figure 5d). We attribute the observation of a larger shift in part to the fact that Msh2–Msh6 recognizes 6-MI:G essentially as a symmetric G:G mismatch and does not exhibit a preferred orientation in binding [43].

In the sequence context of the 6-MI_A:A and A:A_6-MI duplexes, the probe fluorescence emission provides some insight regarding whether Phe 432 stabilization occurs 5′ or 3′ to the mismatch site. With A:A_6-MI we observe a spectral blue shift >10 nm suggesting the Phe is inserted between adenine and the 6-MI on the 3′ side (Figure 5e). The 6-MI_A:A duplex, however, exhibits a smaller blue shift of approximately 5 nm (Figure 5f), potentially reflecting reduced Phe intercalation 5′ of the mismatch. 

Alternatively, Phe intercalation on the 3′ side of the mismatch (that is 6-MI_A:A Phe432) could contribute to the moderate blue shift observed by promoting increased rigidity and stacking interactions. Together, the larger spectral shift observed for the A:A_6-MI substrate and the reduction in dynamics upon protein binding (Figure 4 and Figure 5) point to preferred Phe stabilization on the 3′ side of the mismatch.

### 2.5. Msh2-Msh6-Induced DNA Bending Measured by Förster Resonance Energy Transfer

We also investigated whether the amount of protein-induced bending of mismatched DNA correlated with Msh2–Msh6 binding affinity. We performed steady-state and time-resolved FRET measurements on four representative DNA substrates, spanning a low to high affinity range. Specifically, A:A-, G:T- and T:T-mismatch-containing duplexes as well as G:C homoduplex DNA were measured as the high, moderate and low binding affinity substrates for Msh2–Msh6. We introduced the probes at positions 3 on the Watson strand and 7 on the Crick strand (Table S6) on the DNA duplexes, where the distance between the donor and acceptor dyes is 25 bps or ~85 Å if a canonical B-form duplex is considered. Alexa Fluor 594 and Alexa Fluor 647 were used as the donor and acceptor pair, which has a relatively long Förster radius (R_0_), of 75 Å. The Förster radius is the distance at which energy transfer is 50% efficient and can be used to estimate the effective measurable distance range of this dye pair, which is 37.5 to 150 Å (Table 2).

**Figure 6:**
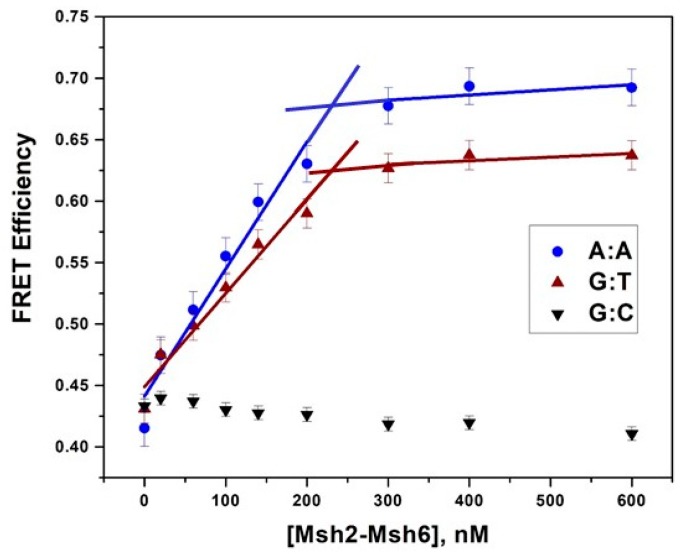
Steady-state Förster Resonance Energy Transfer (FRET) efficiency change upon Msh2–Msh6 binding for A:A and G:T duplex DNA is indicative of protein-induced bending and yields a 1:1 binding stoichiometry. A 200 nM concentration of either A:A, G:T mismatched or G:C homoduplex DNA doubly-labeled with Alexa Fluor 594 and Alexa Fluor 647 was incubated with increasing concentrations of Msh2–Msh6 up to 600 nM. FRET efficiencies were calculated as described in the text. A FRET efficiency increase of ~0.21–0.27 is observed upon protein binding and yields a binding stoichiometry of 1:1 protein:DNA for the A:A and G:T mismatched DNA. The G:C duplex exhibits a slight *decrease* in FRET efficiency upon protein binding, suggesting that a relatively straight conformation is maintained in the presence of protein.

In the absence of Msh2-Msh6 the steady-state FRET efficiencies of all the DNA substrates were found to be approximately 0.43 (Figure 6), which corresponds to a distance between the dye pairs of approximately 78 ± 15 Å (Table 2). Although the orientation of the dyes introduces considerable error into the distance measurement [50], FRET efficiencies and relative efficiency changes of the same or similar substrates are determined with greater accuracy. For the homoduplex substrate, this efficiency and distance suggests that the majority of the population is in a straight conformation, as expected. Interestingly, approximately the same efficiency (0.37–0.42 ± 0.01) is obtained for the mismatch-containing substrates, suggesting that these substrates in the absence of protein also approximate the structure of canonical B-DNA and are in a relatively straight conformation (Table 2).

**Figure 7:**
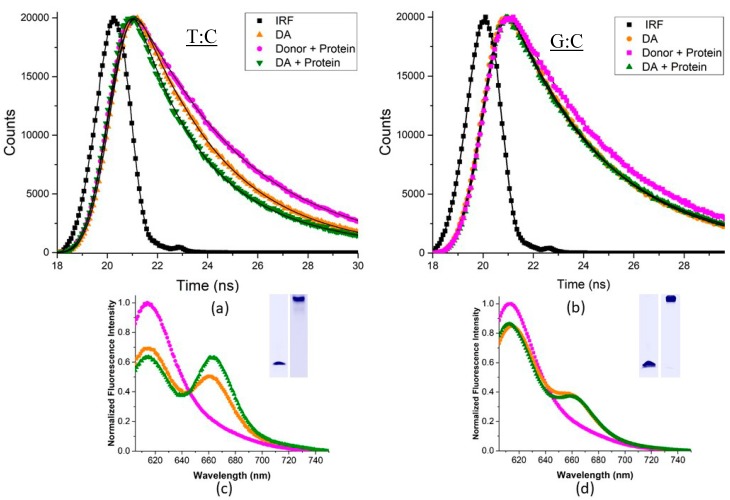
Time-resolved lifetime decays of mismatched substrates. Shorter lifetimes are detected in the presence of protein implying an increase in FRET efficiency for mismatched substrates such as (**a**) T:C mismatched duplex, but not (**b**) G:C duplex DNA upon Msh2–Msh6 binding. (**c**,**d**): Steady-state spectra of the same samples post irradiation show similar trends in FRET efficiency change. The samples were also run on a gel (inset) to show the formation of the protein-DNA complex and lack of sample degradation. 200 nM labeled DNA was used with sufficient Msh2–Msh6 protein to ensure >90% protein-bound DNA. The instrument response function is shown in black. Decays were collected using an excitation wavelength of 590 nm and monitoring emission at 620 nm.

We took a deeper look into these findings with time-resolved FRET measurements of the same DNA substrates and fluorescence dye pairs (Figure 7 and Figure S3). In these measurements, the ratio of fluorescence lifetimes of the donor and donor–acceptor samples are compared to determine the FRET efficiency. Donor-only substrates exhibit only one lifetime of ~4.3 ns (Table 2), which is consistent with reported values for the free dye lifetime [51], while a shorter lifetime is observed for donor–acceptor substrates, consistent with some energy transfer (Table 2). In the absence of protein, all the donor–acceptor labeled substrates examined exhibit lifetimes in the 2.3–2.7 ns range. These lifetimes correspond to energy transfer efficiencies of 0.37–0.47 (±0.013), which is in line with our steady state FRET efficiencies that indicated these substrates are primarily in a relatively straight conformation.

In the steady state experiments, increasing concentrations of Msh2–Msh6 led to an increase in FRET efficiency for the A:A, G:T and T:T mismatched duplexes, signifying a shorter distance between the dyes a consequence of protein-induced bending (Figure 6 and Table 2). For the A:A and G:T substrates, the binding was measured under stoichiometric conditions. As a result of the relatively low affinity of the T:T and T:C substrates, measurements were obtained under equilibrium binding conditions with >90% of the DNA in complex with protein. Under these saturating binding conditions, we measured FRET efficiencies in the 0.57–0.69 range for all the substrates with changes in efficiency ranging from 0.2–0.28 (Table 2). Given the similarities in DNA sequences and the identical placement of the probes, these efficiencies are directly comparable and strongly suggest that Msh2–Msh6 induces the same amount of bending regardless of binding affinity and mismatched substrate.

Time-resolved fluorescence measurements yield similar results for the protein-bound complexes (>90% Msh2–Msh6-bound DNA; Figure 7), where the shorter lifetime component in the donor–acceptor labeled substrates decreases an additional 0.4–0.5 ns upon protein binding. As a control we also examined the donor-only substrates in the presence of protein and observed little to no effect on the lifetime of the donor dye (Figure S4). Thus, the observation of a shorter lifetime does not arise from adventitious quenching from the protein but rather an increase in energy transfer, which we attribute to protein-induced bending. We did not detect any change in the long lifetime component in keeping with assignment to donor only molecules. Steady state spectra of the same samples (Figure 7c,d) confirm the observed changes in lifetime, and the gel insets demonstrate that the DNA was completely bound and remained intact during the experiment.

## 3. Discussion

### 3.1. Msh2–Msh6 Induces the Same Amount of Bending in Different Mismatched Duplexes but Not Homoduplex DNA

X-ray co crystal structures of *E. coli* and *T. aquaticus* MutS or human MutSα with mismatched DNA imply a model of protein-induced bending upon recognition of a mismatch site [16,17,19,20,21]. The time-resolved and steady-state fluorescence results presented in this study strongly align with this model. In the co-crystal structures the +T or G:T mismatched duplex DNA is sharply distorted, with a bend angle ranging from 45° to 60° [16,17,19]. Single-molecule FRET studies have found that in the absence of protein the G:C homoduplex and G:T mismatched duplex DNA mainly sample low FRET efficiency or relatively straight conformations [52]; and upon binding *T. aquaticus* MutS, the G:T mismatched duplex but not the G:C homoduplex exhibits a new high-efficiency FRET or bent population [43,52]. Similarly, in our study with *S. cerevisiae* Msh2–Msh6, the initial FRET efficiency values for mismatched and homoduplexes free of protein were the same; whereas upon protein binding, efficiency values of mismatched DNA increased while the G:C homoduplex DNA did not, indicative of a protein-bound unbent state. Our steady-state and time-resolved FRET analysis extends previous measurements by examining and comparing efficiencies of high and low affinity binding substrates with and without protein (Figure 6 and Figure 7). We used the law of cosines and time-resolved efficiencies to model the DNA bend angle in these complexes with a single, smooth bend in the center [53] and estimate an average bend angle of 66°, consistent with previous crystallography studies [16,17,19]. Although our determination of efficiency values has little experimental error (±0.013), the rotational flexibility of the dyes introduces considerable error into our absolute distance determination (±15 Å) [50] which also influences our calculation of the degree of bending (±10°). Nonetheless, the relatively constant efficiency changes measured for all mismatches are robust, and suggest that both high affinity and low affinity mismatched substrates experience the same amount of protein-induced bending.

An MD study of duplex DNA containing either matched or mismatched bps has suggested that the free energy needed to bend homoduplex DNA is higher than for bending mismatched DNA [27]. Bending free energies are estimated to range from 2.5–6.0 kcal/mol with the highest cost associated with matched bp because of the relative lack of flexibility. Since our measurements indicate that *S. cerevisiae* Msh2–Msh6 induces the same degree of bending regardless of the flexibility or rigidity of the mismatched duplex DNA, we can estimate the free energy cost for bending from the differences in *K*_D_ values for Msh2–Msh6 binding. For the 6-MI_T:T duplex relative to the 6-MI_T:G duplex, we obtain a free energy difference (ΔΔG) of 0.65 kcal/mol and similarly, 0.87 kcal/mol for the 6-MI_T:C duplex. These ΔΔG values are smaller (−0.4–0.7 kcal/mol) than those obtained by comparing the *K*_D_ values of 6-MI:T with 6-MI:C (ΔΔG = 1.3 kcal/mol), which is consistent with the T:T and T:C mismatches exhibiting greater flexibility and dynamics relative to a G:C bp, although they are not as flexible and dynamic as the other high affinity mismatches such as G:T. Thus, our findings support the previous proposal that the differences in binding free energy result from increased flexibility of mismatched relative to matched bps, potentially facilitating recognition of a variety of mismatches by Msh2–Msh6. The higher binding free energies observed for less dynamic mismatches, such as T:T and T:C, result in part from reduced bending propensities as proposed from the MD results [27] and are on the order of 1 kcal/mol.

### 3.2. Msh2–Msh6 Binding Affinity Depends on Mismatch Type

Our measurements of *S. cerevisiae* Msh2–Msh6 binding affinity for seven different mismatched duplexes using the internal fluorescent probe 6-MI improve current understanding of how the Msh2–Msh6 protein complex recognizes a DNA mismatch within the context of paired bases, when the post replicative error rate is approximately 1 mismatch in 10 million bps [1,12]. Our solution binding results indicating binding affinity depends on mismatch type are consistent with previous *E. coli* MutS, *S. cerevisiae* Msh2–Msh6 and human hMsh2–hMsh6 affinity measurements performed with EMSA and surface plasmon resonance (SPR) methods [28,29,30]. These studies also reported that the relative affinity of these interactions depends on the sequence context of the mismatch or IDL site [28,30] and that *E. coli* MutS and hMsh2–hMsh6 bind weakly to pyrimidine:pyrimidine mismatches (C:C, C:T and T:T), which are inefficiently repaired in vivo [29,30,54,55]. Thus, recognition of and high affinity binding to a mismatch are important factors in the efficiency of DNA repair.

Although our findings generally agree with previously reported trends [28,29], our solution fluorescence anisotropy measurements yield a different binding affinity order for mismatches (Figure 1). We observed higher binding affinities for A:A and A:G mismatched bps relative to G:T and obtained the following order of equilibrium binding affinity for different mismatched bps: A:A ≈ A:G > G:G > A:C ≈ G:T > T:T > T:C (Table 1). We attribute the differences in measured binding affinities to differences in the protein used and methodologies applied, particularly when comparing solution to gel binding assays [41,42]. Additionally, the differences in protein binding affinities reported for the same mismatch by different research groups can also result from the sequence context of the mismatch site, specifically the flanking bases [28,30,56,57]. Such effects potentially play a role in our finding that Msh2–Msh6 binds to G:T mismatches with slightly different affinities in different sequence contexts (13.7 nM for 6-MI:T duplex and 25.9 nM for T:G_6-MI duplex in which the 6-MI is located 3′ to the mismatch on the Crick strand). We detect similar differences for the G:A mismatch, where a *K*_D_ value of 25.8 nM is obtained for the 6-MI:A duplex and 9.0 nM is obtained for the 6-MI_A:G mismatched duplex. Thermodynamic measurements indicate that the contribution of a single mismatch to duplex stability is strongly dependent on its nearest-neighbor bases, and mismatches such as A:G and A:C are more strongly influenced than other mismatches [58,59,60]. The relatively high purine content surrounding our mismatch site, particularly at the 3′ position could facilitate the recognition of the A:G and A:C mismatches relative to other mismatches [30]. As previously reported, purine:purine and purine:pyrimidine mismatches are recognized with greater affinity by Msh2–Msh6 or MutSα relative to pyrimidine:pyrimidine mismatches [28,29,30,61]. The tight binding interaction between MutS or Msh2–Msh6 and G:T or +T mismatches partially accounts for the high repair efficiency of these mismatches observed in both prokaryotic and eukaryotic MMR systems [31,33,55].

### 3.3. Msh2-Msh6 Binding Affinity Correlates with Enhanced Dynamics at or next to a Mismatch Site

**Figure 8:**
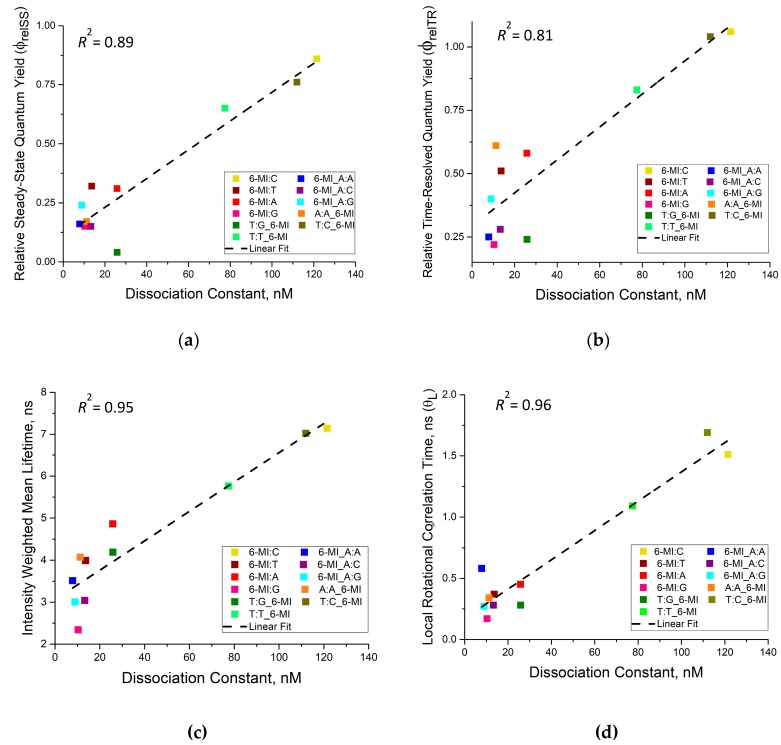
Linear relationships between (**a**) steady state quantum yield, (**b**) time-resolved quantum yield, (**c**) mean excited-state lifetime (τ_m_) and (**d**) local rotational correlation time (*θ*_L_) of 6-MI and the dissociation constant (*K*_D_) of Msh2-Msh6 for various mismatched duplexes. Mismatched bps with low quantum yields (ϕ), shorter mean lifetimes (τ_m_ < 5 ns) and shorter local rotational correlation times (*θ*_L_ < 0.5 ns), indicative of high local flexibility and dynamics, are well-recognized by Msh2–Msh6 (*K*_D_ ≤ 25 nM).

Analysis of steady-state and time-resolved fluorescence parameters, such as relative fluorescence quantum yields (Figure 2), excited-state lifetimes (Figure 3) and rotational correlation times (Figure 4), provide insight into the dynamics of the 6-MI probe incorporated at or next to different mismatched bps. For high-affinity substrates (*K*_D_ ≤ 25 nM), namely A:A, A:G, A:C, G:G and G:T, we observed a significant reduction in steady-state and time-resolved relative quantum yields, consistent with a shorter mean lifetime (Figure 3), and we also detected faster local rotational correlation times, *θ*_𝐿_, through fluorescence anisotropy decay measurements (Figure 4). The changes in all of these parameters indicate 6-MI experiences a greater amount of dynamic quenching from adjacent bases and the backbone in these mismatched DNA substrates relative to canonical B-DNA. We also find that all measures of dynamic motion linearly correlate with Msh2–Msh6 affinity for the mismatched bp (Figure 8). Notably, the *θ*_𝐿_ values of mismatched substrates with *K*_D_ values less than or equal to 25 nM (6-MI:X, 6-MI_A:X, A:A_6-MI and T:G_6-MI) are relatively fast, which suggests that the local dynamics of the mismatch and the bases surrounding the mismatch facilitate Msh2–Msh6 binding and recognition. In contrast, *θ*_𝐿_ values of moderate to low affinity T:T and T:C mismatched substrates are two- to three-fold longer. Moreover, T:T and T:C mismatches exhibit longer mean lifetimes with higher relative quantum yields that are comparable to that of the 6-MI:C homoduplex (Figure 8), suggesting restricted local dynamics at these mismatched sites similar to that of a matched bp. This finding provides experimental support for previous MD simulation results suggesting that T:C and C:C mismatches exhibit breathing frequencies similar to canonical bps [36] and is consistent with base and hydrodynamic measurements using a Hoechst dye bound in the minor groove, which have also shown that the T:T mismatch resembles canonical B-DNA [62].

Mismatch incorporation minimally perturbs the global conformation of B-form DNA, as reported by X-ray crystal and NMR solution structures; however dynamic and structural changes have been detected in the vicinity of a mismatch [34,59,63,64]. A NMR spectroscopy study coupled with MD simulations showed that mismatched bps exhibited a higher level of DNA breathing frequency or spontaneous conformational fluctuations relative to canonical A:T and G:C bps [36]. The presence of mismatches and unpaired bases also perturbs stacking with adjacent bases [34,63]. In a previous study, Nag and co-workers detected enhanced dynamics of 2-AP when placed adjacent to either a G:T mismatched bp or a +T site using time-resolved fluorescence spectroscopy [35]. This study only compared G:T and C:C mismatches and observed increased dynamics for the G:T and not C:C mismatch. The current results improve and extend the previous work as we detect enhanced dynamics in all well-recognized (*K*_D_ ≤ 25 nM) mismatched bps, not just G:T. The comparable results from these studies affirm that the observed dynamics are not specific to a particular method or probe but are a feature of well-recognized mismatches (Figure 8).

Previous results point to a model in which mismatch dynamics play a role in Msh2–Msh6 recognition and consequent repair efficiency [30,38]. Our study also supports this model and further reveals that Msh2–Msh6 binding affinity increases linearly with all measures of DNA dynamics, with higher binding affinities observed for dynamic mismatched bp (*θ*_L_ < 0.6 ns) (Figure 8). Interestingly, correlations between bending propensity and binding affinity were examined previously [27] and the finding was that bending propensity is not sufficient to explain the experimental binding preferences. Single-molecule measurements have shown that MutS [24] or Msh2–Msh6 [65] slides along the DNA contour using a one-dimensional diffusion process, presumably searching for any changes in structure or dynamics caused by the presence of a mismatch. Importantly, our results suggest that the dynamics of the mismatched bp and surrounding area on the duplex potentially trigger recognition and binding (Figure 8). Thus, the low Msh2–Msh6 binding affinity and repair efficiency of pyrimidine:pyrimidine mismatches potentially arises from the relatively ′stiff′ nature of these mismatches which masks their identity to Msh2–Msh6 during its 1-D search.

### 3.4. Msh2–Msh6 Stabilizes the Mismatch Upon Binding

The increase in the local rotational correlation time and mean lifetime of 6-MI in the 6-MI:X (X = T, A, G) duplexes strongly support a model in which the mismatch site is stabilized upon *S. cerevisiae* Msh2–Msh6 binding. This protein-induced stabilization is also observed in the T:G_6-MI and A:A_6-MI substrates. Similarly, our previous work reporting the dynamics of 6-MI located 3′ to a +T IDL showed a two- to three-fold increase in the local rotational correlation time of the probe upon protein binding [40]. This stabilization effect, however, is not observed when the 6-MI probe is located 5′ to the mismatch, such as in the 6-MI_A:X duplexes. We attribute this increase in *θ*_𝐿_ and mean lifetime to stabilization of 6-MI caused by the stacking of Msh6 Phe 432 and the H-bonding of Msh6 Glu 434 to the mismatched or unpaired thymine, as observed in the Msh2–Msh6•G:T or Msh2–Msh6•+T co-crystal structures [19]. Notably, the T:T_6-MI and T:C_6-MI mismatched duplexes do not exhibit protein-induced stabilization of the 6-MI probe. In these substrates, presumably the 6-MI probe is already stabilized in the absence of protein through formation of a wobble bp. We speculate that the possibility of forming a tautomer [66,67,68,69,70] that can make more H-bonds than a wobble bp could further stabilize these mismatches and reduce the dynamics of the mispaired base.

**Figure 9:**
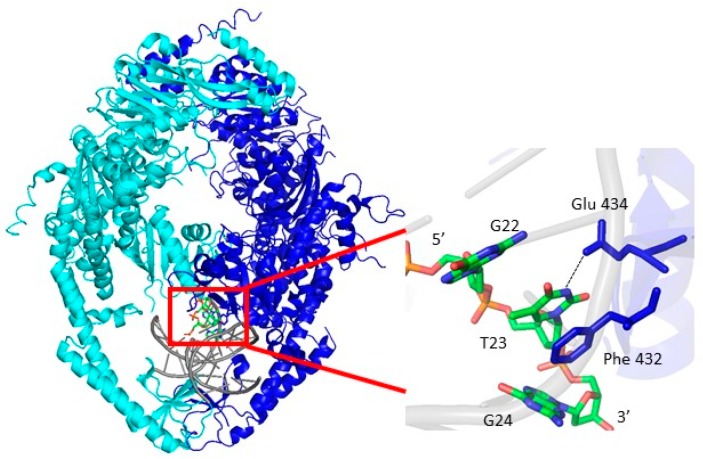
Structural details of the mismatch binding domain of hMsh6 interacting with G:T mismatched DNA as determined by X-ray crystallography. Stabilization of the thymine in the G:T mismatched bp through stacking of the conserved Phe 432 from the 3′ side and hydrogen bonding of the conserved Glu 434 is highlighted. Phe 432 and Glu 434 are shown in blue, the nucleic acid residues are shown in elemental colors and the Msh2 subunit is shown in teal. The structure was generated using PyMol and the PDB file 2O8B.

In the Msh2–Msh6-G:T or Msh2–Msh6•+T co-crystal structures, Phe 432 stacks with the mismatched or unpaired thymine on the 3′ side. We find this interaction directly stabilizes the mismatch site and the adjacent bases on the 3′ side, but not those on the 5′ side (Figure 9). The importance of this Phe residue has been established in *T. aquaticus* MutS [71] and *S. cerevisiae* Msh2–Msh6 [72], where a Phe to Ala mutation yields a protein complex that no longer stably binds to mismatched DNA. Chemical footprinting experiments performed with the G:T mismatched duplex and *T. aquaticus* MutS also indicate that the MutS-bound G:T mismatched duplex is more protected on the 3′ side, and in an MD study a partial opening of the 5′ base next to the mismatch is observed [27,73]. Similarly, greater stabilization of the 2-AP probe located 3′ to a G:T mismatch is observed upon MutS binding, indicative of a similar directional stabilization effect [35]. Thus, our observation of probe stabilization 3′ but not 5′ of the mismatch, coupled with spectral shifts occurring only when the probe is 3′ of the mismatch, are consistent with directional stabilization by Msh2–Msh6. Finally, we suggest that the difference in local base dynamics and protein stabilization of the mismatch observed between high-affinity and low-affinity substrates is a mechanism for MutS proteins to recognize the mismatch bp in the context of matched DNA and correlates with efficiency of repair.

## 4. Materials and Methods

### 4.1. S. cerevisiae Msh2–Msh6 Purification from E. coli Cells

*S. cerevisiae* Msh2–Msh6 proteins were over-expressed and purified from *E.coli* cells, using a previously established protocol [74].

### 4.2. Oligonucleotides

Crude single stranded DNAs were purchased from Integrated DNA Technologies (IDT, Coralville, IA). The DNA strands were purified using 20% denaturing polyacrylamide gels containing urea as previously described [40]. For a 34-mer DNA strand, 4–5 h of electrophoresis at 400 V is needed for the DNA to travel 15 cm in the gel, prior to identification of the band by UV shadowing. The DNA band was then excised from the gel. Electroelution (Schlechier and Schuell, Dassel, Germany) at 100 V for 8 h was used to extract the DNA from the gel pieces followed by dialysis against ddH2O. A Vivaspin 500 centrifugal concentrator (Vivaproducts Inc., Littleton, MA, USA) with a 3 kDa MWCO was used to concentrate the sample, and the DNA concentration was determined by measuring the ultraviolet (UV) absorbance at 260 nm using the manufacturer supplied extinction coefficient.

HPLC-purified DNA strands containing pteridine nucleoside analog 6-methylisoxanthopterin (6-MI) were purchased from Fidelity Systems (Gaithersburg, MD, USA), and were used without further purification (Table S1).

Duplex DNAs were annealed by mixing equal molar amounts of complementary strands in an annealing buffer (10 mM Tris-Cl pH 8.0, 1 mM EDTA, 300 mM NaCl). The samples were heated up to 90 °C for 5 min and then slow cooled to room temperature over 8–10 h.

The oligonucleotides synthesized with an amine-modified C6 linker on the 5′ end were purchased and purified as described above. The fluorescent probes used for labeling were the amine-reactive succinimidyl esters purchased from Thermo Fisher Scientific (Waltham, MA, USA), including 5-carboxyfluorescein succinimidyl ester (FAM), 5-carboxytetramethylrhodamine succinimidyl ester (TAMRA), Alexa Fluor 594 succinimidyl ester and Alexa Fluor 647 succinimidyl ester. The amine-modified oligonucleotide sequences are listed in Table S6. Before labeling, the oligonucleotides were concentrated to 25 µg/µL in ddH2O using a Vivaspin^®^ 500 centrifugal concentrator with a MWCO of 3 kDa. Labeling was performed following the manufacturer′s protocol [75].

### 4.3. Steady-State Quantum Yield

The steady-state quantum yields were determined as described [76]. The calculation of quantum yield was based on the fluorescence and absorbance of five samples of different concentrations, using the equation given below,

(1) $$\phi_{x}=\;\phi_{ST}\left(\frac{Grad_{x}}{Grad_{ST}}\right)\left(\frac{\eta_{x}^{2}}{\eta_{ST}^{2}}\right)$$

where the subscripts 𝑆𝑇 and 𝑋 represent standard and sample respectively, 𝜙 is the fluorescence quantum yield, 𝐺𝑟𝑎𝑑 is the gradient from the plot of integrated fluorescence intensity vs. absorbance and 𝜂 denotes the refractive index of the solvent. For the Alexa Fluor dyes, FAM in 0.1 M NaOH was used as a standard with a quantum yield value of 0.79 [77]. For 6-MI incorporated duplex DNAs, the steady-state quantum yields were determined relative to 6-MI monomer, which has a quantum yield of 0.7 [39,44,45].

Absorbance measurements were performed with a Beckman Coulter DU-650 spectrophotometer (Beckman Coulter Inc., Brea, CA, USA), with sample absorbance readings between 0.1–1 at the maximum absorption wavelength in a quartz cell of 1 cm path length. Fluorescence intensity readings were recorded using Horiba FluoroMax^®^-4 spectrometer (Horiba). Labeled oligomers were diluted to ensure the maximum counts per second were less than 2 × 10^6^. Samples were contained in 3 mm square quartz cuvettes, and the scanning rate was 1 nm/pt, with an integration time of 1 s/pt. The excitation polarizer was set to 0°and emission polarizer was set to 54.7° to eliminate any polarization bias. Spectral analysis and integrations were performed with GRAMS/AI™ spectroscopy software (Thermo Fisher Scientific).

### 4.4. Fluorescence Emission Spectra

The fluorescence emission spectra were acquired using Horiba FluoroMax^®^-4 spectrometer (Horiba). Spectra were collected using an excitation wavelength of 320 nm and emission was monitored from 380 to 525 nm at a resolution of 0.5 nm/pt with an integration of 5 s/pt. The excitation and emission band pass were set to 6 and 2 nm respectively. For the protein-bound samples, a concentration of 20 nM 6-MI labeled DNA was used with a sufficiently high protein concentration to ensure >90% of the DNA was bound. Msh2–Msh6-DNA complexes were incubated in a 10 mM sodium phosphate, pH 7.5, 100 mM NaCl, 5 mM MgCl_2_ binding buffer for 3 min at 10 °C before each measurement.

### 4.5. Electrophoretic Mobility Shift Assay

Electrophoretic mobility shift assays (EMSA) were performed between the purified *S. cerevisiae* Msh2-Msh6 protein with each of the different DNA substrates. A 4.5% non-denaturing acrylamide gel was prepared in 40 mM Tris-Borate and 1 mM EDTA (TBE buffer) with 5% glycerol. 10 nM DNA substrates with 0.0–1.0 µM Msh2–Msh6 were incubated for 30 min in 20 mM Tris-Cl pH 7.5, 100 mM NaCl, 5 mM MgCl_2_, 2% ficoll at 4 °C. The total volume of each sample was 20 µL. The gels were pre-run at a constant voltage of 100 V for 1 h before sample loading. Samples were loaded and a voltage of 100 V was applied until the DNA had migrated approximately two-thirds down the gel. The gels were stained with SYBR Green I Nucleic Acid Gel Stain (Thermo Fisher Scientific) for 10 min and detected using a Typhoon 9200 imager (GE Healthcare Life Sciences, Little Chalfont, UK). An excitation wavelength of 488 nm and a band pass emission filter of 520 nm were used for gel imaging.

### 4.6. Fluorescence Anisotropy Equilibrium Binding Experiments

Fluorescence anisotropy experiments of Msh2–Msh6 binding to different DNA substrates were performed by titrating protein into 10 nM DNA using 5 mm square quartz or glass cuvettes. The binding buffer contained 10 mM sodium phosphate, pH 7.5, 100 mM NaCl, 5 mM MgCl_2_. The Msh2–Msh6 protein was incubated for 3 min at 10 °C in the sample holder before each measurement. For the 6-MI labeled DNA, the excitation wavelength was 340 nm and emission was monitored at 430 nm, both with a 4 nm bandpass. Measurements were performed with a 1 min integration time with the polarizers set to either vertical/vertical (*V*/*V*) or vertical/horizontal (*V*/*H*) positions for the excitation and emission polarizers. Background and buffer contributions were subtracted and at least 3 independent measurements were performed. The binding curve was analyzed assuming a 1:1 binding interaction of protein and DNA with the following equation:

(2) $$r=r_{i}+\left(r_{f}-r_{i}\right)\times \left(\frac{\left({\left[D\right]}_{T}+\;{\left[P\right]}_{T}+K_{D}\right)-\;\sqrt{{\left({\left[D\right]}_{T}+\;{\left[P\right]}_{T}+K_{D}\right)}^{2}-4{\left[D\right]}_{T}{\left[P\right]}_{T}}}{2{\left[D\right]}_{T}}\right)$$

where r refers to the anisotropy measured at a certain protein concentration, 𝑟𝑖 and 𝑟𝑓 represent the initial and final anisotropy values, [D]*_T_* and [P]*_T_* represent the total molar concentration of the DNA and protein respectively, and *K*_D_ is the dissociation constant. The binding parameters were obtained using a non-linear least squares fitting method with Origin 8.0 (OriginLab, Northampton, MA, USA) and the quality of the fit was assessed by the χ^2^ parameter and visual inspection of the residuals.

### 4.7. Fluorescence Lifetime and Anisotropy Decays

Fluorescence lifetime measurements were performed using a Photon Technology International (PTI) TimeMaster™ (Horiba) time-correlated single-photon counting (TCSPC) instrument. A sample of 200 nM ds DNA containing 6-MI with and without Msh2–Msh6 was measured in 5 mm square quartz cuvettes. A BDL-375-SMC 375 nm pulsed picosecond diode laser (Becker & Hickl GmbH, Berlin, Germany) (repetition rate: 1 MHz; average power <1 mW) was used as the excitation light source. Fluorescence emission was detected at 460 nm with a 450 nm cut-off filter. Both excitation and emission slits were set to 15 mm. The excitation and emission polarizers were set to 0° and 54.7°, respectively, for the lifetime measurement. Both 0° (*V*/*V*) polarization and 90° (*V*/*H*) polarization data were acquired for the anisotropy decay analysis. For *V*/*V* decay, a total of 20000 counts was collected in the peak channel for each trial. The *V*/*H* decay was collected for the same amount of time as the *V*/*V* decay to account for any polarization bias in detection.

The instrument response function (IRF) was recorded at 375 nm using diluted LUDOX^®^ AS-40 colloidal silica (Sigma-Aldrich, St. Louis, MO, USA). The full width at half-maximum (FWHM) of the IRF was ~250 ps spanning 14 channels. The 6-MI incorporated duplex DNA mixed with Msh2–Msh6 was measured under conditions where at least 85% of the DNA was complexed to protein. The samples were incubated at 10 °C for 10 min with stirring before data acquisition.

The deconvoluted fluorescence intensity decays were analyzed using a sum of exponentials as follows,

(3) $$I\left(t\right)=\;\sum_{i}a_{i}e^{\left(\frac{-t}{\tau_{i}}\right)}$$

where 𝐼(𝑡) represents the intensity at time 𝑡, 𝛼_𝑖_ is proportional to the fractional population with a lifetime 𝜏_𝑖_. Note that ∑𝛼_𝑖_ is normalized to unity. The fluorescence intensity decay of 6-MI incorporated samples was fit to a multi-exponential function using an iterative reconvolution method with Globals WE software (Laboratory for Fluorescence Dynamics, Irvine, CA, USA).

Time-dependent anisotropy decays of the 6-MI incorporated duplex DNA were recorded in the absence and presence of Msh2–Msh6. The anisotropy decay 𝑟(𝑡) was calculated by measured *V*/*V* and *V*/*H* intensity decay as described previously, and the data was fit to a sum of exponential decay terms,

(4) $$r\left(t\right)=r_{0}\;\sum_{i}g_{i}e^{\left(-\frac{t}{\theta_{i}}\right)}\;$$

where 𝑟_0_ represents the limiting anisotropy without rotational diffusion, 𝑔𝑖 represents the fractional amplitude associated with each rotational correlation time 𝜃_𝑖_. The sum of fractional amplitudes was normalized such that $\underset{i}{\sum }g_{i}=1$ For two components this equation becomes:

(5) $$r\left(t\right)=r_{0}\left(\beta_{1}e^{\left(\frac{-t}{\theta_{1}}\right)}+\beta_{2}e^{\left(\frac{-t}{\theta_{2}}\right)}\right)$$

where 𝜃_1_ and 𝜃_2_ represent the two correlation times resolved by fitting the decays and *β*_1_ represents the fractional amplitude associated with the rotational correlation time 𝜃_1_. Similar to the fluorescence intensity decays, the anisotropy decays were fit to a double-exponential model using iterative deconvolution, yielded the corresponding 𝜃_1_ and 𝜃_2_ values. The quality of the data fitting was determined by the reduced χ2 value (ranging from 1.05 to 1.32) and visual inspection of the weighted residuals. Two rotational correlation times representing the fast local motion of the fluorophore 6-MI (*θ*_L_) and the global rotational motion of the duplex DNA (*θ*_R_) are used to describe this experimental system. We assume the local motion of the fluorophore occurs independently of the tumbling motion of the DNA, and rewrite Equation (5) as,

(6) $$r\left(t\right)=r_{0}\left(\beta_{1}e^{\left(\frac{-t}{\theta_{L}}\right)}+\left(\beta_{2}\right)\right)e^{\left(\frac{-t}{\theta_{R}}\right)}$$

The fast component of the decay (𝜃_1_) is influenced by both the local dynamics of 6-MI and the overall rotational motion of the DNA,

(7) $$\frac{1}{\theta_{1}}=\;\frac{1}{\theta_{L}}+\;\frac{1}{\theta_{R}}$$

The slow component of the decay (𝜃_2_) is equated to the global rotational time of the DNA (𝜃_R_), as the contribution of the fast motion to this decay is much smaller, given the shorter time scale, so,

𝜃_2_ = 𝜃_R_ (8)

The values of 𝜃_L_ and 𝜃_R_ can be calculated using Equations (7) and (8). Data were analyzed using Globals WE software (Laboratory for Fluorescence Dynamics).

### 4.8. Steady-State and Time-Resolved Förster Resonance Energy Transfer (FRET) Assays and Analysis

The distance between dye pairs attached to DNA at positions 3 on the Watson strand and 7 on the Crick strand on the 34 DNA duplex was obtained in the absence and presence of Msh2–Msh6 using FRET. The energy transfer efficiency was calculated using the fluorescence intensity of the donor in the presence (𝐹_𝐷𝐴_) and absence (𝐹_𝐷_) of acceptor, in the following equation,

(9) $$E=1-\frac{F_{DA}}{F_{D}}$$

Similarly, the lifetime of the donor in the presence (𝜏_𝐷𝐴_) and absence (𝜏_𝐷_) of acceptor was also used to calculate the transfer efficiency,

(10) $$E=1-\frac{\tau_{DA}}{\tau_{D}}$$

To determine the degree of protein-induced bending through steady-state assays, the duplex DNA was end labeled with the FRET dye pair Alexa Fluor 594 and Alexa Fluor 647. An excitation wavelength of 590 nm was used for Alexa Fluor 594 and the emission was recorded from 605 to 750 nm. The direct excitation of Alexa Fluor 647 was carried out by exciting at 648 nm and the emission was recorded from 660 to 750 nm. The measurements were performed in 5 mm square quartz cuvettes at 200 nM DNA concentration, with slits widths set to 5 and 5 nm for excitation and emission on a Horiba FluoroMax-4 spectrometer (Horiba). The scan rate was 1 nm/pt with a 1 sec integration time for each point; the excitation polarizer was set to 0° and emission polarizer was set to 54.7°. Msh2–Msh6 was incubated in a 10 mM sodium phosphate, pH 7.5, 100 mM NaCl, 5 mM MgCl_2_ binding buffer for 3 min at 10 °C before each measurement.

The time-resolved FRET data were acquired using DNA samples with the same labeling scheme and in the same buffer conditions as described for the steady-state measurements. The time-resolved FRET measurements were performed using the same instrument described above except a Horiba 590 nm LED laser (EL590; rep rate = 180 Khz) was used to excite the samples. Emission was detected at 620 nm using a 600 nm cut-off filter and a time window of 75 ns. Excitation and emission slits were set to a 25 nm spectral bandpass and the intensity decay data were collected to a maximum of 20,000 counts in the peak channel. Msh2–Msh6 was incubated in a 10 mM sodium phosphate, pH 7.5, 100 mM NaCl, 5 mM MgCl_2_ binding buffer for 3 min at 10 °C before each measurement.

The experimentally-determined energy transfer efficiency 𝐸 is used to calculate the distance *R* between dye pairs using the following equation,

(11) $$R={\left(\frac{1}{E}-1\right)}^{\frac{1}{6}}*\;R_{0}$$

where *R*_0_ is defined as the distance at which the transfer is 50% efficient or the Förster distance and can be calculated using the following equation:

(12) $$R_{0}=0.211\times {\left(\kappa^{2}\eta^{-4}\Phi_{D}J\left(\lambda \right)\right)}^{\frac{1}{6}}\;(\mathrm{in}\;Å)$$

where κ^2^ is the factor indicating the relative orientation of the transition dipole moments of the donor and acceptor chromophores and for freely rotating molecules, this value is assumed to be 2/3 [47]. Other parameters are *η*, the index of refraction, Φ*_D_*, the quantum yield of the donor and *J(λ)*, the overlap integral of the emission spectrum of the donor and the absorption spectrum of the acceptor [47]. R_0_ values were determined using the protein and labeled DNA under the binding conditions.

Once the distance *R* between the donor and acceptor is known, protein-induced bending of the duplex DNA can be determined using the law of cosines, assuming the bending is a smooth deformation in the middle of the duplex DNA [53]. The bending angle (180–θ)° can be calculated using the following equation,

(13) $$cos\theta =\;\frac{a^{2}+\;b^{2}-\;R^{2}}{2ab}$$

where a and b represent the distances between the dye pairs to the center of the bend, respectively. Other functions for determining the bend angle [78] yield comparable results within the error of our measurement.

## 5. Conclusions

The current study confirms previous findings that *S. cerevisiae* Msh2–Msh6 exhibits differential binding affinities for different types of mismatches. In addition to extending our study to seven different mismatches, we also monitor global bending through FRET and the local dynamic properties of these mismatches through the fluorescent nucleoside analog, 6-MI. As our measurements reveal, all mismatches are bent to the same degree and we find that binding affinity is linearly correlated with local dynamics and not DNA bending. By studying multiple mismatches in addition to G:T, we determined that local bp dynamics are a critical element of Msh2–Msh6 recognition. Our findings, which monitored both global DNA structure and local DNA dynamics, suggest an induced fit rather than conformational capture mechanism governs initial mismatch binding and recognition. Moreover, our spectroscopic results indicate the Phe 432 residue consistently stacks with the mismatch on the 3′ side. Thus, this study, through a combination of spectroscopic and biochemical investigations, provides new insights into initial identification and recognition of mismatched bp by Msh2–Msh6. Specifically, we find that Msh2–Msh6 employs a common strategy to probe for and interact with any non-Watson-Crick site, and the efficiency of recognition and repair of a particular mismatch depends on the local dynamics of the site. The current study also demonstrates the utility of the 6-MI probe for investigating base-specific dynamics of DNA in protein–DNA interactions.

## Figures and Tables

**Table 1:** Equilibrium binding affinity of *S. cerevisiae* Msh2-Msh6 with mismatched duplex DNA measured by fluorescence spectroscopy.

DNA Substrate	*K*_D_^1^ (nM)	*K*_D_^2^ (nM)	*K*_D_^3^ (nM)
6-MI:C	121.5 ± 12.1	-	106.5 ± 13.9
6-MI:T	13.7 ± 3.4	-	13.3 ± 3.9
6-MI:A	25.8 ± 12.3	-	-
6-MI:G	10.4 ± 5.8	-	15.6 ± 3.4
T:G_6-MI	25.9 ± 1.2	-	-
T:T_6-MI	77.5 ± 20.0	-	-
T:C_6-MI	112.6 ± 18.7	-	-
A:A_6-MI	11.3 ± 3.1	10.4 ± 2.4	-
6-MI_A:A	7.9 ± 2.0	19.1 ± 3.5	-
6-MI_A:G	9.0 ± 2.8	11.8 ± 2.8	-
6-MI_A:C	13.3 ± 3.6	17.1 ± 4.3	-
+T_6-MI	16.0 ± 2.9	18.1 ± 2.6	-

^1^*K*_D_ values determined from fluorescence anisotropy measurements of Msh2–Msh6 binding to 6-MI labeled duplex DNA. ^2^ *K*_D_ values determined from the fluorescence intensity change in the same binding experiment. ^3^ *K*_D_ values determined from fluorescence anisotropy binding isotherm of Msh2–Msh6 with duplex DNA with the same sequence labeled with Alexa Fluor 594 or Alexa Fluor 647. The average data is reported with a standard deviation from three independent measurements.

**Table 2:** Steady-state (SS) and time-resolved ^1^ (TR) Förster Resonance Energy Transfer Measurement Parameters ^2^.

Mismatch Type	Sample ^3^	α_1_	τ_1_	α_2_	τ_2_	χ^2^	TR E_FRET_^4^ ±0.013	TR ΔE_FRET_	TR R ^5^ (Å) ±15 Å	TR Angle ^6^ (°) ±10°	SS E_FRET_^4^	SS ΔE_FRET_	SS R ^5^ (Å) ±15Å	SS Angle ^6^ (°) ±10°
T:T	D	1.00	4.28			1.25	0.47	0.14	77	70	0.42	0.27	80	79
	DA	0.82	4.28	0.18	2.26	1.18								
T:T with Msh2–Msh6	D	1.00	4.36			1.29	0.62		70		0.69		66	
	DA	0.80	4.36	0.20	1.66	1.24								
G:T	D	1.00	4.28			1.19	0.37	0.15	82	59	0.43	0.21	79	73
	DA	0.84	4.28	0.16	2.69	0.98								
G:T with Msh2–Msh6	D	1.00	4.33			1.17	0.53		74		0.64		69	
	DA	0.90	4.33	0.10	2.05	1.01								
A:A	D	1.00	4.25			1.23	0.42	0.18	80	68	0.42	0.28	80	78
	DA	0.82	4.25	0.18	2.47	1.03								
A:A with Msh2–Msh6	D	1.00	4.31			1.38	0.60		71		0.69		66	
	DA	0.90	4.31	0.10	1.72	1.12								
T:C	D	1.00	4.28			1.25	0.38	0.22	80	68	0.37	0.20	82	64
	DA	0.52	4.28	0.48	2.62	0.85								
T:C with Msh2–Msh6	D	1.00	4.29			1.13	0.60		71		0.57		72	
	DA	0.64	4.28	0.36	1.72	1.95								
G:C	D	1.00	4.29			1.20	0.45	0.00	78		0.43	−0.02	79	
	DA	0.78	4.29	0.22	2.37	1.21								
G:C with Msh2–Msh6	D	1.00	4.41			1.42	0.45		78		0.41		80	
	DA	0.82	4.41	0.18	2.42	0.97								

^1^ Time-resolved data was fit to a sum of exponentials (Equation (3)) as described in the text. Original data shown in Figure 7 and Figure S4. ^2^ All parameters reported are derived from at least three independent experiments. ^3^ D = donor only molecules. DA = doubly-labeled donor–acceptor molecules. ^4^ All FRET efficiencies were calculated as described in the text using Equations (9) and (10). ^5^ Distance or R values were calculated as described in the text using Equations (11) and (12) with an R_0_ value of 75.5 Å. ^6^ Bend angles were calculated using the law of cosines (Equation (13)) as described in the text.

## Supplementary Materials

Supplementary materials can be found at https://www.mdpi.com/1422-0067/20/17/4271/s1.

## Abbreviations

MMR	Mismatch repair
6-MI	6-Methylisoxanthopterin
Msh	MutS Homolog
IDL	Insertion/deletion loop
AFM	Atomic force microscopy
FRET	Förster resonance energy transfer
sm-FRET	Single-molecule Förster resonance energy transfer
MD	Molecular dynamics
bp	Base pair
EMSA	Electrophoretic mobility shift assay
𝜙_𝑟𝑒𝑙__SS_	Relative steady-state fluorescence quantum yield
𝜙_𝑟𝑒𝑙__TR_	Relative time-resolved fluorescence quantum yield
IRF	Instrument response function
2-AP	2-Aminopurine
SPR	Surface plasmon resonance
TCSPC	Time-correlated single-photon counting
FWHM	Full width at half-maximum
FAM	5-Carboxyfluorescein succinimidyl ester
TAMRA	5-Carboxytetramethylrhodamine succinimidyl ester
LED	Light-emitting diode
MWCO	Molecular weight cut off

## References

[1] Kunkel T.A., Erie D.A. (2005). DNA mismatch repair. Annu. Rev. Biochem..

[2] Jiricny J. (2006). MutLalpha: at the cutting edge of mismatch repair. Cell.

[3] Spies M., Fishel R. (2015). Mismatch repair during homologous and homeologous recombination. Cold Spring Harbor Perspect. Biol..

[4] Kunkel T.A., Erie D.A. (2015). Eukaryotic mismatch repair in relation to DNA replication. Annu. Rev. Genet..

[5] Boland C.R., Lynch H.T. (2013). The history of Lynch syndrome. Fam. Cancer.

[6] Lynch H.T., Snyder C.L., Shaw T.G., Heinen C.D., Hitchins M.P. (2015). Milestones of Lynch syndrome: 1895–2015. Nat. Rev. Cancer.

[7] Lebbink J.H., Drost M., de Wind N. (2016). DNA mismatch repair: from biophysics to bedside. DNA Repair.

[8] Heinen C.D. (2016). Mismatch repair defects and Lynch syndrome: The role of the basic scientist in the battle against cancer. DNA Repair.

[9] Fishel R. (2015). Mismatch repair. J. Biol. Chem..

[10] Modrich P. (2006). Mechanisms in eukaryotic mismatch repair. J. Biol. Chem..

[11] Groothuizen F.S., Sixma T.K. (2016). The conserved molecular machinery in DNA mismatch repair enzyme structures. DNA Repair.

[12] Hingorani M.M. (2016). Mismatch binding, ADP-ATP exchange and intramolecular signaling during mismatch repair. DNA Repair.

[13] Reyes G.X., Schmidt T.T., Kolodner R.D., Hombauer H. (2015). New insights into the mechanism of DNA mismatch repair. Chromosoma.

[14] Polyzos A.A., McMurray C.T. (2017). Close encounters: Moving along bumps, breaks, and bubbles on expanded trinucleotide tracts. DNA Repair.

[15] Schmidt M.H., Pearson C.E. (2016). Disease-associated repeat instability and mismatch repair. DNA Repair.

[16] Lamers M.H., Perrakis A., Enzlin J.H., Winterwerp H.H., de Wind N., Sixma T.K. (2000). The crystal structure of DNA mismatch repair protein MutS binding to a G x T mismatch. Nature.

[17] Obmolova G., Ban C., Hsieh P., Yang W. (2000). Crystal structures of mismatch repair protein MutS and its complex with a substrate DNA. Nature.

[18] Natrajan G., Lamers M.H., Enzlin J.H., Winterwerp H.H., Perrakis A., Sixma T.K. (2003). Structures of Escherichia coli DNA mismatch repair enzyme MutS in complex with different mismatches: a common recognition mode for diverse substrates. Nucleic Acids Res..

[19] Warren J.J., Pohlhaus T.J., Changela A., Iyer R.R., Modrich P.L., Beese L.S. (2007). Structure of the human MutSalpha DNA lesion recognition complex. Mol. Cell.

[20] Groothuizen F.S., Winkler I., Cristóvão M., Fish A., Winterwerp H.H.K., Reumer A., Marx A.D., Hermans N., Nicholls R.A., Murshudov G.N. (2015). MutS/MutL crystal structure reveals that the MutS sliding clamp loads MutL onto DNA. eLife.

[21] Wang H., Yang Y., Schofield M.J., Du C., Fridman Y., Lee S.D., Larson E.D., Drummond J.T., Alani E., Hsieh P. (2003). DNA bending and unbending by MutS govern mismatch recognition and specificity. Proc. Natl. Acad. Sci. USA.

[22] Tessmer I., Yang Y., Zhai J., Du C., Hsieh P., Hingorani M.M., Erie D.A. (2008). Mechanism of MutS searching for DNA mismatches and signaling repair. J. Biol. Chem..

[23] DeRocco V.C., Sass L.E., Qiu R., Weninger K.R., Erie D.A. (2014). Dynamics of MutS-mismatched DNA complexes are predictive of their repair phenotypes. Biochemistry.

[24] Lee J.B., Cho W.K., Park J., Jeon Y., Kim D., Lee S.H., Fishel R. (2014). Single-molecule views of MutS on mismatched DNA. DNA Repair.

[25] Jeong C., Cho W.K., Song K.M., Cook C., Yoon T.Y., Ban C., Fishel R., Lee J.B. (2011). MutS switches between two fundamentally distinct clamps during mismatch repair. Nat. Struct. Mol. Biol..

[26] Gorman J., Wang F., Redding S., Plys A.J., Fazio T., Wind S., Alani E.E., Greene E.C. (2012). Single-molecule imaging reveals target-search mechanisms during DNA mismatch repair. Proc. Natl. Acad. Sci. USA.

[27] Sharma M., Predeus A.V., Mukherjee S., Feig M. (2013). DNA bending propensity in the presence of base mismatches: implications for DNA repair. J. Phys. Chem. B.

[28] Marsischky G.T., Kolodner R.D. (1999). Biochemical characterization of the interaction between the Saccharomyces cerevisiae MSH2-MSH6 complex and mispaired bases in DNA. J. Biol. Chem..

[29] Brown J., Brown T., Fox K.R. (2001). Affinity of mismatch-binding protein MutS for heteroduplexes containing different mismatches. Biochem. J..

[30] Mazurek A., Johnson C.N., Germann M.W., Fishel R. (2009). Sequence context effect for hMSH2-hMSH6 mismatch-dependent activation. Proc. Natl. Acad. Sci. USA.

[31] Kramer B., Kramer W., Fritz H.J. (1984). Different base/base mismatches are corrected with different efficiencies by the methyl-directed DNA mismatch-repair system of E. coli. Cell.

[32] Dohet C., Wagner R., Radman M. (1985). Repair of defined single base-pair mismatches in Escherichia coli. Proc. Natl. Acad. Sci. USA.

[33] David P., Efrati E., Tocco G., Krauss S.W., Goodman M.F. (1997). DNA replication and postreplication mismatch repair in cell-free extracts from cultured human neuroblastoma and fibroblast cells. J. Neurosci..

[34] Isaacs R.J., Rayens W.S., Spielmann H.P. (2002). Structural differences in the NOE-derived structure of G-T mismatched DNA relative to normal DNA are correlated with differences in (13)C relaxation-based internal dynamics. J. Mol. Biol..

[35] Nag N., Rao B.J., Krishnamoorthy G. (2007). Altered dynamics of DNA bases adjacent to a mismatch: a cue for mismatch recognition by MutS. J. Mol. Biol..

[36] Rossetti G., Dans P.D., Gomez-Pinto I., Ivani I., Gonzalez C., Orozco M. (2015). The structural impact of DNA mismatches. Nucleic Acids Res..

[37] Imhof P., Zahran M. (2013). The effect of a G:T mispair on the dynamics of DNA. PLoS ONE.

[38] Isaacs R.J., Spielmann H.P. (2004). A model for initial DNA lesion recognition by NER and MMR based on local conformational flexibility. DNA Repair.

[39] Hawkins M.E. (2001). Fluorescent pteridine nucleoside analogs: A window on DNA interactions. Cell Biochem. Biophys..

[40] Moreno A., Knee J., Mukerji I. (2012). Applying 6-methylisoxanthopterin-enhanced fluorescence to examine protein-DNA interactions in the picomolar range. Biochemistry.

[41] Fried M., Liu G. (1994). Molecular sequestration stabilizes CAP-DNA complexes during polyacrylamide gel electrophoresis. Nucleic Acids Res..

[42] Hellman L.M., Fried M.G. (2007). Electrophoretic mobility shift assay (EMSA) for detecting protein-nucleic acid interactions. Nat. Protoc..

[43] Cristovao M., Sisamakis E., Hingorani M.M., Marx A.D., Jung C.P., Rothwell P.J., Seidel C.A., Friedhoff P. (2012). Single-molecule multiparameter fluorescence spectroscopy reveals directional MutS binding to mismatched bases in DNA. Nucleic Acids Res..

[44] Hawkins M.E., Pfleiderer W., Balis F.M., Porter D., Knutson J.R. (1997). Fluorescence properties of pteridine nucleoside analogs as monomers and incorporated into oligonucleotides. Anal. Biochem..

[45] Hawkins M.E., Pfleiderer W., Mazumder A., Pommier Y.G., Balis F.M. (1995). Incorporation of a fluorescent guanosine analog into oligonucleotides and its application to a real time assay for the HIV-1 integrase 3′-processing reaction. Nucleic Acids Res..

[46] Moreno A., Knee J.L., Mukerji I. (2016). Photophysical characterization of enhanced 6-methylisoxanthopterin fluorescence in duplex DNA. J. Phys. Chem. B.

[47] Lakowicz J.R. (2006). Principles of Fluorescence Spectroscopy.

[48] Wojtuszewski Poulin K., Smirnov A.V., Hawkins M.E., Balis F.M., Knutson J.R. (2009). Conformational heterogeneity and quasi-static self-quenching in DNA containing a fluorescent guanine analogue, 3MI or 6MI. Biochemistry.

[49] Hawkins M.E. (2008). Fluorescent pteridine probes for nucleic acid analysis. Methods Enzymol..

[50] Ivanov V., Li M., Mizuuchi K. (2009). Impact of emission anisotropy on fluorescence spectroscopy and FRET distance measurements. Biophys. J..

[51] Thermo Fisher Scientific-US, Fluorescence Quantum Yields (QY) and Lifetimes (τ) for Alexa Fluor Dyes-Table 1.5. www.thermofisher.com/us/en/home/references/molecular-probes-the-handbook/tables/fluorescence-quantum-yields-and-lifetimes-for-alexa-fluor-dyes.html.

[52] Sass L.E., Lanyi C., Weninger K., Erie D.A. (2010). Single-molecule FRET TACKLE reveals highly dynamic mismatched DNA-MutS complexes. Biochemistry.

[53] Litke J.L., Li Y., Nocka L.M., Mukerji I. (2016). Probing the ion binding site in a DNA holliday junction using forster resonance energy transfer (FRET). Int. J. Mol. Sci..

[54] Bishop D.K., Andersen J., Kolodner R.D. (1989). Specificity of mismatch repair following transformation of Saccharomyces cerevisiae with heteroduplex plasmid DNA. Proc. Natl. Acad. Sci. USA.

[55] Kramer B., Kramer W., Williamson M.S., Fogel S. (1989). Heteroduplex DNA correction in Saccharomyces cerevisiae is mismatch specific and requires functional PMS genes. Mol. Cell. Biol..

[56] Jones M., Wagner R., Radman M. (1987). Repair of a mismatch is influenced by the base composition of the surrounding nucleotide sequence. Genetics.

[57] Su S.S., Lahue R.S., Au K.G., Modrich P. (1988). Mispair specificity of methyl-directed DNA mismatch correction in vitro. J. Biol. Chem..

[58] Allawi H.T., SantaLucia J. (1997). Thermodynamics and NMR of internal G.T mismatches in DNA. Biochemistry.

[59] Allawi H.T., SantaLucia J. (1998). Nearest neighbor thermodynamic parameters for internal G.A mismatches in DNA. Biochemistry.

[60] Allawi H.T., SantaLucia J. (1998). Nearest-neighbor thermodynamics of internal A.C mismatches in DNA: sequence dependence and pH effects. Biochemistry.

[61] Li G.M. (2008). Mechanisms and functions of DNA mismatch repair. Cell Res..

[62] Shweta H., Singh M.K., Yadav K., Verma S.D., Pal N., Sen S. (2017). Effect of T.T mismatch on DNA dynamics probed by minor groove binders: Comparison of dynamic stokes shifts of hoechst and DAPI. J. Phys. Chem. B.

[63] Brown T., Hunter W.N., Kneale G., Kennard O. (1986). Molecular structure of the G.A base pair in DNA and its implications for the mechanism of transversion mutations. Proc. Natl. Acad. Sci. USA.

[64] Boulard Y., Cognet J.A., Fazakerley G.V. (1997). Solution structure as a function of pH of two central mismatches, C . T and C . C, in the 29 to 39 K-ras gene sequence, by nuclear magnetic resonance and molecular dynamics. J. Mol. Biol..

[65] Gorman J., Chowdhury A., Surtees J.A., Shimada J., Reichman D.R., Alani E., Greene E.C. (2007). Dynamic basis for one-dimensional DNA scanning by the mismatch repair complex Msh2-Msh6. Mol. Cell..

[66] Szymanski E.S., Kimsey I.J., Al-Hashimi H.M. (2017). Direct NMR evidence that transient tautomeric and anionic states in dG.dT form watson-crick-like base pairs. J. Am. Chem. Soc..

[67] Goodman M.F. (2018). Smoking gun for a rare mutation mechanism. Nature.

[68] Kimsey I.J., Szymanski E.S., Zahurancik W.J., Shakya A., Xue Y., Chu C.C., Sathyamoorthy B., Suo Z., Al-Hashimi H.M. (2018). Dynamic basis for dG*dT misincorporation via tautomerization and ionization. Nature.

[69] Kouchakdjian M., Li B.F., Swann P.F., Patel D.J. (1988). Pyrimidine.pyrimidine base-pair mismatches in DNA. A nuclear magnetic resonance study of T.T pairing at neutral pH and C.C pairing at acidic pH in dodecanucleotide duplexes. J. Mol. Biol..

[70] Cornelis A.G., Haasnoot J.H., den Hartog J.F., de Rooij M., van Boom J.H., Cornelis A. (1979). Local destabilisation of a DNA double helix by a T--T wobble pair. Nature.

[71] Yamamoto A., Schofield M.J., Biswas I., Hsieh P. (2000). Requirement for Phe36 for DNA binding and mismatch repair by *Escherichia coli* MutS protein. Nucleic Acids Res..

[72] Bowers J., Sokolsky T., Quach T., Alani E. (1999). A mutation in the MSH6 subunit of the saccharomyces cerevisiae MSH2-MSH6 complex disrupts mismatch recognition. J. Biol. Chem..

[73] Biswas I., Hsieh P. (1997). Interaction of MutS protein with the major and minor grooves of a heteroduplex DNA. J. Biol. Chem..

[74] Antony E., Hingorani M.M. (2003). Mismatch recognition-coupled stabilization of Msh2-Msh6 in an ATP-bound state at the initiation of DNA repair. Biochemistry.

[75] Thermo Fisher Scientific Amine Reactive Probe Labeling Protocol. https://www.thermofisher.com/us/en/home/references/protocols/cell-and-tissue-analysis/labeling-chemistry-protocols/amine-reactive-probe-labeling-protocol.html.

[76] Horiba Scientific A Guide to Recording Fluorescence Quantum Yields. http://www.horiba.com/fileadmin/uploads/Scientific/Documents/Fluorescence/quantumyieldstrad.pdf.

[77] Umberger J.Q., LaMer V.K. (1945). The kinetics of diffusion controlled molecular and ionic reactions in solution as determined by measurements of quenching of fluorescence. J. Am. Chem. Soc..

[78] Wojtuszewski K., Mukerji I. (2003). HU binding to bent DNA: A fluorescence resonance energy transfer and anisotropy study. Biochemistry.

