# Supplementary material for: Mismatch Recognition by Saccharomyces cerevisiae Msh2-Msh6: Role of Structure and Dynamics
Source: Int J Mol Sci. 2019 Aug 31;20(17):4271. doi: 10.3390/ijms20174271 (PMC6747400; doi:10.3390/ijms20174271)
Supplement: Supplementary file 1 [file ijms-20-04271-s001.pdf]

International Journal of
Molecular Sciences

Supporting Materials

Mismatch Recognition by *Saccharomyces cerevisiae* Msh2-Msh6: Role of Structure and Dynamics

Yan Li, Zane Lombardo, Meera Joshi, Manju M. Hingorani and Ishita Mukerji *

Molecular Biology and Biochemistry Department, Molecular Biophysics Program, Wesleyan University; Middletown, CT 06459, USA

* Correspondence: imukerji@wesleyan.edu; Tel.: +1-860-685-2422

Received: 7 August 2019; Accepted: 27 August 2019; Published: 31 August 2019

1. Supplementary Materials

1.1. Supplementary Figures

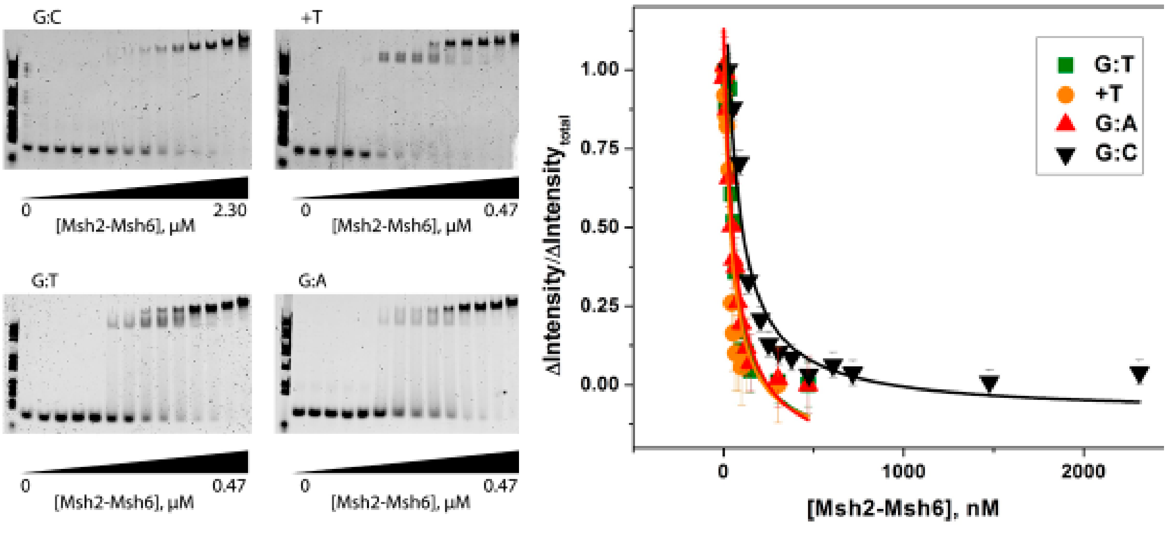

(**a**) (**b**)

Figure S1. Binding of Msh2-Msh6 protein to homoduplex and mismatched duplex DNA measured by EMSA. (**a**) Msh2-Msh6 binding to 34 basepair homoduplex DNA and +T, G:T and G:A mismatched duplex DNA. The concentration of *S. cerevisiae* Msh2-Msh6 was increased from 0 to 0.47 μM and the DNA concentration was held constant at 10 nM for the unlabeled G:C/+T/G:T/G:A duplex substrates. For the unlabeled G:C homoduplex DNA the Msh2-Msh6 concentration was increased up to 2.30 μM. The multiple bands observed at higher concentrations of protein are attributed to protein-DNA complexes. Non-specific binding and/or changes in DNA conformation lead to the observation of more than one band; (**b**) The intensity of the free DNA bands decreases as a function of Msh2-Msh6 concentration indicative of protein binding. Analysis of the decrease (as described in Materials and Methods) yields a K_{Dapp} for Msh2-Msh6 binding to G:C, +T, G:T and G:A substrates.

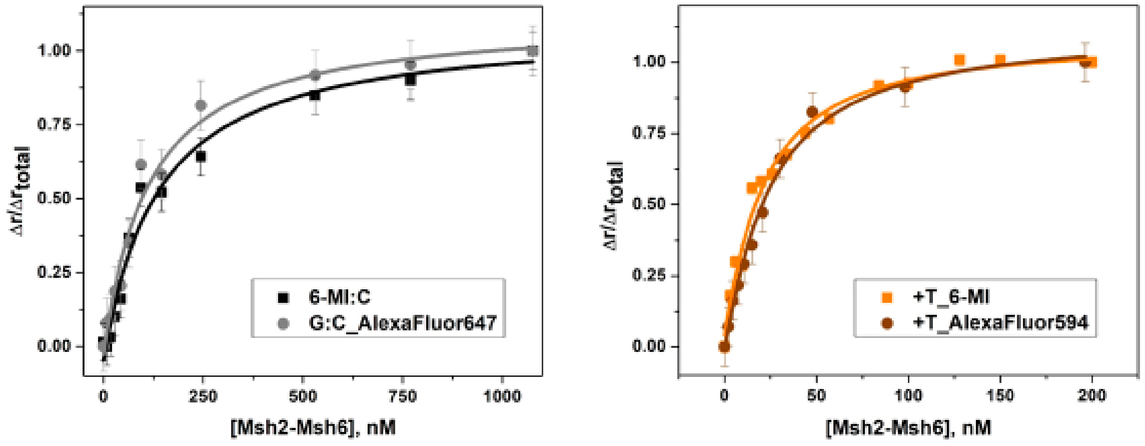

(a) (b)

Figure S2. Equilibrium binding curves of Msh2-Msh6 with homoduplex DNA and +T duplex DNA measured with fluorescence anisotropy using 6-MI and externally labeled duplexes demonstrate that Msh2-Msh6 does not differentially recognize 6-MI. (**a**) Titration of 10 nM 6-MI:C (black square) and G:C_AF647 (gray circle) with Msh2-Msh6 gives K_D values of 121.5 ± 12.1 nM and 92.1 ± 13.9 nM, respectively; (**b**) Msh2-Msh6 binding to 10 nM of +T_6-MI (orange square) and +T_AF594 (brown circle) yields K_D values of 16.0 ± 2.9 nM and 18.1 ± 2.6 nM respectively, as measured by fluorescence anisotropy. The K_D values obtained with the internally- and externally-labeled substrates are within error of each other, confirming that 6-MI does not perturb the binding of Msh2-Msh6 to DNA.

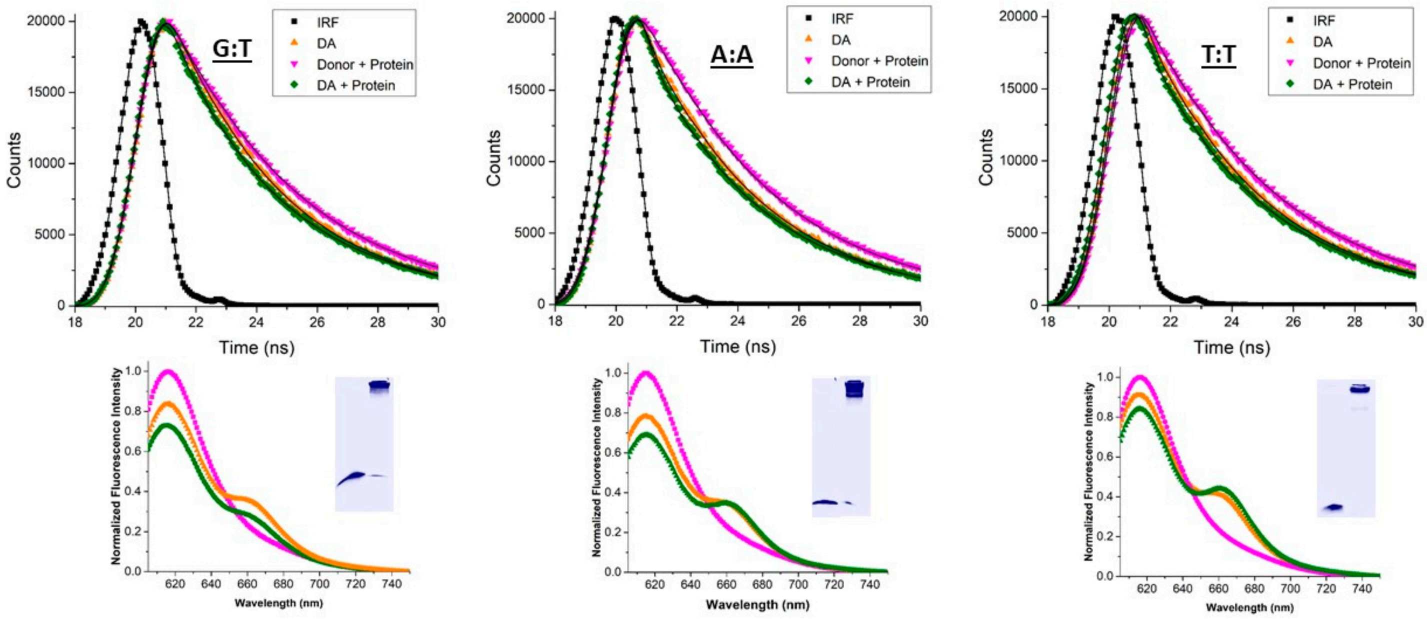

(a) (b) (c)

Figure S3. Protein-binding increases FRET efficiency for the (**a**) G:T (**b**) A:A, and **(c)** T:T mismatched substrates. Top: Fluorescence intensity decays which show for all three mismatches that protein binding leads to a decrease in donor lifetime. Bottom: Steady-state fluorescence spectra confirm the increase in energy transfer through a decrease in donor fluorescence with protein bound. The samples were also run on a gel (inset) to show the formation of the protein-DNA complex and lack of degradation post irradiation. To investigate the bound complex, sufficient protein was added to the 200 nM labeled DNA to ensure >90% of the DNA was bound. The instrument response function is shown in black. Decays were collected with 590 nm excitation and 620 nm emission. 20,000 peak channel counts were collected in two independent experiments for each substrate.

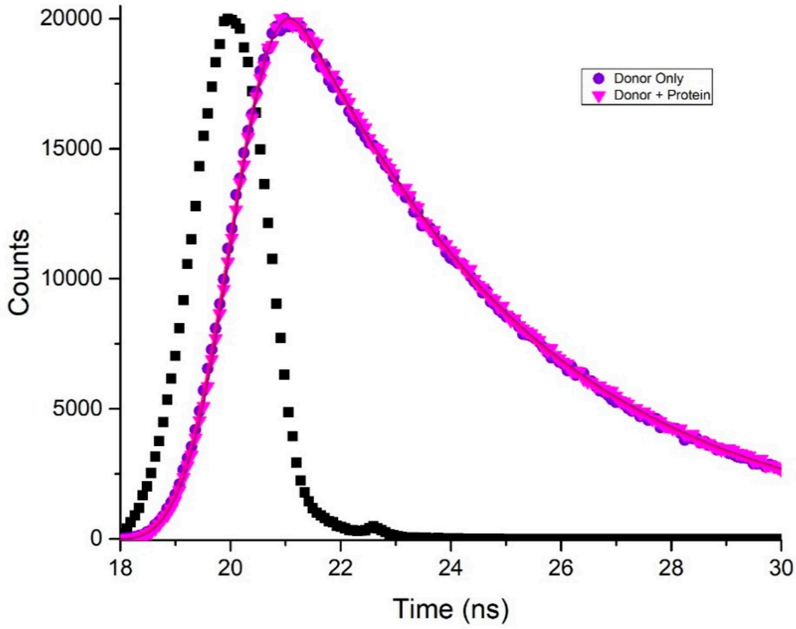

Figure S4. Time-resolved fluorescence decays of the G:T donor only duplex show no shift in populations upon addition of Msh2-Msh6. G:T mismatched DNA labeled with the donor dye, free (purple) and bound to protein (magenta). The decays yield comparable fitting parameters (Table 2) consistent with little to no effect on donor fluorescence from protein binding. The instrument response function is shown in black. Decays were collected at an emission wavelength of 620 nm with 590 nm excitation. The decays were analyzed using a multi-exponential function as described in the Materials and Methods.

1.2. Supplementary Tables

Table S1. Sequence[1] of 6-MI incorporated oligonucleotides used in this study.

Name	Sequence
6-MI:C	**5′ TAT GCA GTC ACT ATF AAT CAA CTA CTT AGA TGGT 3′**<br>**3′ ATA CGT CAG TGA TAC TTA GTT GAT GAA TCT ACCA 5′**
6-MI:T	**5′ TAT GCA GTC ACT ATF AAT CAA CTA CTT AGA TGGT 3′**<br>**3′ ATA CGT CAG TGA TAT TTA GTT GAT GAA TCT ACCA 5′**
6-MI:A	**5′ TAT GCA GTC ACT ATF AAT CAA CTA CTT AGA TGGT 3′**<br>**3′ ATA CGT CAG TGA TAA TTA GTT GAT GAA TCT ACCA 5′**
6-MI:G	**5′ TAT GCA GTC ACT ATF AAT CAA CTA CTT AGA TGGT 3′**<br>**3′ ATA CGT CAG TGA TAG TTA GTT GAT GAA TCT ACCA 5′**
T:G_6-MI	**5′ TAT GCA GTC ACT ATF AAT CAA CTA CTT AGA TGGT 3′**<br>**3′ ATA CGT CAG TGA TGC TTA GTT GAT GAA TCT ACCA 5′**
T:T_6-MI	**5′ TAT GCA GTC ACT ATF AAT CAA CTA CTT AGA TGGT 3′**<br>**3′ ATA CGT CAG TGA TTC TTA GTT GAT GAA TCT ACCA 5′**
T:C_6-MI	**5′ TAT GCA GTC ACT ATF AAT CAA CTA CTT AGA TGGT 3′**<br>**3′ ATA CGT CAG TGA TCC TTA GTT GAT GAA TCT ACCA 5′**
6-MI_A:G	**5′ TAT GCA GTC ACT ATF AAT CAA CTA CTT AGA TGGT 3′**<br>**3′ ATA CGT CAG TGA TAC GTA GTT GAT GAA TCT ACCA 5′**

6-MI_A:C	**5′ TAT GCA GTC ACT ATF AAT CAA CTA CTT AGA TGGT 3′**<br>**3′ ATA CGT CAG TGA TAC CTA GTT GAT GAA TCT ACCA 5′**
6-MI_A:A	**5′ TAT GCA GTC ACT ATF AAT CAA CTA CTT AGA TGGT 3′**<br>**3′ ATA CGT CAG TGA TAC ATA GTT GAT GAA TCT ACCA 5′**
A:A_6-MI	**5′ TAT GCA GTC ACT ATG AAT CAA CTA CAT AGA TGGT 3′**<br>**3′ ATA CGT CAG TGA TAC TTA GTT GAT FAA TCT ACCA 5′**
+T_6-MI	**5′ TAT GCA GTC ACT ATF AAT CAA CTA CTT AGA TGGT 3′**<br>**3′ ATA CGT CAG TGA T_C TTA GTT GAT GAA TCT ACCA 5′**

[1]F designates 6-MI. The pentamer sequence containing 6-MI is highlighted in yellow. 6-MI base pairs are colored in red and mismatched base pairs/IDL are colored in green.

Table S2. The apparent dissociation constants K_{Dapp} obtained for the four substrates from Figure S1.

DNA substrate	K_{Dapp}, nM[1]
G:C	66 ± 24
+T	33 ± 14
G:T	35 ± 15
G:A	45 ± 16

[1] The error reported is the standard deviation from at least 3 different experiments.

Table S3. Steady-state[1] and time-resolved[2] fluorescence quantum yields of 6-MI containing duplex DNA.

Sequence Name	ϕ_{rel}SS (±0.05)	ϕ_{rel}TR (±0.05)
6-MI	1	1
6-MI:C	0.86	1.06
6-MI:T	0.32	0.51
6-MI:A	0.31	0.58
6-MI:G	0.15	0.22
T:G_6-MI	0.04	0.24
T:T _6-MI	0.65	0.83
T:C_6-MI	0.76	1.04
A:A_6-MI	0.17	0.61
6-MI_A:A	0.16	0.25
6-MI_A:G	0.24	0.40
6-MI_A:C	0.15	0.28

[1]The steady state quantum yields, ϕ_{rel}SS, were measured relative to 6-MI monomer and were determined from three independent measurements. [2]The time-resolved quantum yields (ϕ_{rel}TR) were determined from the fluorescence lifetime measurements reported in Table S4 and are reported relative to the lifetime of 6-MI monomer.

Table S4. Time-resolved fluorescence intensity decay parameters[1] of 6-MI with and without Msh2-Msh6.

Sequence Name	α_1 (±0.01)	τ_1 (ns) (±0.03)	α_2 (±0.01)	τ_2 (ns) (±0.07)	α_3 (±0.01)	τ_3 (ns) (±0.04)	Mean Lifetime (ns) (±0.03)

6-MI[a]	-	-	-	-	1	6.57	6.57
6-MI:C	-	-	0.10	3.47	0.90	7.33	7.14
6-MI:C with Msh2-Msh6	-	-	0.11	3.18	0.89	7.21	7.00
6-MI:T	0.17	1.23	0.79	3.58	0.04	8.95	3.99
6-MI:T with Msh2-Msh6	0.28	0.92	0.65	3.42	0.07	7.84	4.02
6-MI:A	0.24	1.38	0.67	4.04	0.09	9.08	4.86
6-MI:A with Msh2-Msh6	0.24	1.15	0.65	3.94	0.11	8.86	5.05
6-MI:G	0.72	0.96	0.24	2.09	0.04	6.27	2.34
6-MI:G with Msh2-Msh6	0.58	0.71	0.32	2.18	0.10	6.64	3.46
T:G _6-MI	0.61	0.30	0.29	2.38	0.10	7.07	4.19
T:G_6-MI with Msh2-Msh6	0.50	0.40	0.37	2.47	0.13	7.88	4.86
T:T_6-MI	-	-	0.34	3.67	0.66	6.40	5.76
T:T_6-MI with Msh2-Msh6	0.07	1.58	0.40	3.97	0.53	6.27	5.45
T:C_6-MI	-	-	0.09	3.07	0.91	7.18	7.02
T:C_6-MI with Msh2-Msh6	-	-	0.11	2.95	0.89	7.13	6.92
6-MI_A:G	0.18	1.14	0.78	2.73	0.04	6.87	3.00
6-MI_A:G with Msh2-Msh6	0.22	1.33	0.71	3.10	0.07	6.48	3.46
6-MI_A:C	0.42	0.60	0.50	2.20	0.08	6.16	3.04
6-MI_A:C with Msh2-Msh6	0.41	0.68	0.52	2.21	0.07	6.22	2.96
6-MI_A:A	0.57	0.59	0.37	2.28	0.06	7.61	3.51
6-MI_A:A with Msh2-Msh6	0.40	0.67	0.33	2.96	0.27	6.56	4.87
A:A_6-MI	0.25	0.63	0.47	2.95	0.28	5.44	4.07
A:A_6-MI with Msh2-Msh6	0.17	0.98	0.67	2.88	0.16	6.15	3.82

[1]Fluorescence decays were fit to a sum of discrete exponentials:

$$I\,(t) = \sum_i a_i e^{\left(\frac{-t}{\tau_i}\right)} \quad (1)$$

Standard deviations from fitting are given in the parentheses and result from three independent experiments.

[a]6-MI monomer data taken from Moreno, Knee et al. 2012.

Table S5. Time-resolved fluorescence anisotropy decay parameters[1] of 6-MI containing mismatched duplexes, with and without Msh2-Msh6

Duplex Substrate	β_1 (±0.02)	θ_L (ns) (±0.06)	β_2 (±0.02)	θ_R (ns) (±0.9)
6-MI:C	0.10	1.51	0.90	19.9
6-MI:C with protein	0.12	1.53	0.88	24.8
6-MI:T	0.10	0.37	0.90	15.7
6-MI:T with protein	0.15	1.53	0.85	28.9
6-MI:A	0.10	0.45	0.90	16.2
6-MI:A with protein	0.13	1.93	0.87	27.6
6-MI:G	0.10	0.17	0.90	10.0

6-MI:G with protein	0.22	1.70	0.78	29.1
T:G_6-MI	0.21	0.28	0.79	10.4
T:G_6-MI with protein	0.28	2.02	0.72	121.9
T:T_6-MI	0.08	1.09	0.92	18.3
T:T_6-MI with protein	0.08	1.10	0.92	25.0
T:C_6-MI	0.10	1.69	0.90	19.2
T:C_6-MI with protein	0.10	1.65	0.89	21.1
6-MI_A:G	0.06	0.27	0.94	15.3
6-MI_A:G with protein	0.06	0.37	0.94	48.4
6-MI_A:C	0.10	0.28	0.90	9.5
6-MI_A:C with protein	0.07	0.23	0.93	18.8
6-MI_A:A	0.12	0.58	0.88	16.1
6-MI_A:A with protein	0.22	0.22	0.78	67.4
A:A_6-MI	0.09	0.34	0.91	10.4
A:A_6-MI with protein	0.05	1.48	0.95	32.2

[1]Anisotropy decay data were fit to the following expression:

$$r(t) = r_0[\beta_1 e^{\left(\frac{-t}{\theta_L}\right)} + \beta_2] e^{\left(\frac{-t}{\theta_R}\right)} \quad (2)$$

as described in the Materials and Methods. Standard deviations from fitting are given in the parentheses and result from three independent experiments.

Table S6. Sequence of oligonucleotides labeled[1] with external probes in this study.

Name	Sequence
G:C_AF647	**AF647/5AmMC6/TAT GCA GTC ACT ATG AAT CAA CTA CTT AGA TGGT 3′**<br>**3′ ATA CGT CAG TGA TAC TTA GTT GAT GAA TCT ACCA 5′**
+T_AF594	**AF594/5AmMC6/TAT GCA GTC ACT ATG AAT CAA CTA CTT AGA TGGT 3′**<br>**3′ ATA CGT CAG TGA T_C TTA GTT GAT GAA TCT ACCA 5′**
G:T_AF594	**AF594/5AmMC6/TAT GCA GTC ACT ATG AAT CAA CTA CTT AGA TGGT 3′**<br>**3′ ATA CGT CAG TGA TAT TTA GTT GAT GAA TCT ACCA 5′**
FRET_G:C<br>(Doubly)	**AF594**<br>**5′ TA/iAmC6T/ GCA GTC ACT ATG AAT CAA CTA CTT A GA TGGT 3′**<br>**3′ AT A CGT CAG TGA TAC TTA GTT GAT GAA /iAmMC6T/CT ACCA 5′**<br>**AF647**
FRET_A:A<br>(Doubly)	**AF594**<br>**5′ TA/iAmC6T/ GCA GTC ACT ATG AAT CAA CTA CTT A GA TGGT 3′**<br>**3′ AT A CGT CAG TGA TAC ATA GTT GAT GAA /iAmMC6T/CT ACCA 5′**<br>**AF647**

FRET_A:G (Doubly)	**AF594**<br>**5′ TA/iAmC6T/ GCA GTC ACT ATG AAT CAA CTA CTT A GA TGGT 3′**<br>**3′ AT A CGT CAG TGA TAC GTA GTT GAT GAA /iAmMC6T/CT ACCA 5′**<br>**AF647**
FRET_T:G (Doubly)	**AF594**<br>**5′ TA/iAmC6T/ GCA GTC ACT ATG AAT CAA CTA CTT A GA TGGT 3′**<br>**3′ AT A CGT CAG TGA TGC TTA GTT GAT GAA /iAmMC6T/CT ACCA 5′**<br>**AF647**
FRET_T:T (Doubly)	**AF594**<br>**5′ TA/iAmC6T/ GCA GTC ACT ATG AAT CAA CTA CTT A GA TGGT 3′**<br>**3′ AT A CGT CAG TGA TTC TTA GTT GAT GAA /iAmMC6T/CT ACCA 5′**<br>**AF647**

[1]The fluorescent probe is highlighted in yellow. Mismatched base pairs are colored in green. 5AmMC6 and iAmC6T stand for 5′ amino modifier C6 and internal amino modifier C6 dT respectively.
